# Generation and Characterization of a Cell Type-Specific, Inducible Cre-Driver Line to Study Olfactory Processing

**DOI:** 10.1523/JNEUROSCI.3076-20.2021

**Published:** 2021-07-28

**Authors:** Anzhelika Koldaeva, Cary Zhang, Yu-Pei Huang, Janine Kristin Reinert, Seiya Mizuno, Fumihiro Sugiyama, Satoru Takahashi, Taha Soliman, Hiroaki Matsunami, Izumi Fukunaga

**Affiliations:** ^1^Sensory and Behavioural Neuroscience Unit, Okinawa Institute of Science and Technology Graduate University, Okinawa, Japan, 904-0495; ^2^Laboratory Animal Resource Center, Tsukuba University, Ibaraki, Japan, 305-8577; ^3^Department of Molecular Genetics and Microbiology and Department of Neurobiology, Duke University, Durham, North Carolina, 27710

**Keywords:** CRISPR, olfaction, scRNA-seq

## Abstract

In sensory systems of the brain, mechanisms exist to extract distinct features from stimuli to generate a variety of behavioral repertoires. These often correspond to different cell types at various stages in sensory processing. In the mammalian olfactory system, complex information processing starts in the olfactory bulb, whose output is conveyed by mitral cells (MCs) and tufted cells (TCs). Despite many differences between them, and despite the crucial position they occupy in the information hierarchy, Cre-driver lines that distinguish them do not yet exist. Here, we sought to identify genes that are differentially expressed between MCs and TCs of the mouse, with an ultimate goal to generate a cell type-specific Cre-driver line, starting from a transcriptome analysis using a large and publicly available single-cell RNA-seq dataset (Zeisel et al., 2018). Many genes were differentially expressed, but only a few showed consistent expressions in MCs and at the specificity required. After further validating these putative markers using ISH, two genes (i.e., *Pkib* and *Lbdh2*) remained as promising candidates. Using CRISPR/Cas9-mediated gene editing, we generated Cre-driver lines and analyzed the resulting recombination patterns. This indicated that our new inducible Cre-driver line, *Lbhd2-CreERT2*, can be used to genetically label MCs in a tamoxifen dose-dependent manner, both in male and female mice, as assessed by soma locations, projection patterns, and sensory-evoked responses *in vivo*. Hence, this is a promising tool for investigating cell type-specific contributions to olfactory processing and demonstrates the power of publicly accessible data in accelerating science.

**SIGNIFICANCE STATEMENT** In the brain, distinct cell types play unique roles. It is therefore important to have tools for studying unique cell types specifically. For the sense of smell in mammals, information is processed first by circuits of the olfactory bulb, where two types of cells, mitral cells and tufted cells, output different information. We generated a transgenic mouse line that enables mitral cells to be specifically labeled or manipulated. This was achieved by looking for genes that are specific to mitral cells using a large and public gene expression dataset, and creating a transgenic mouse using the gene editing technique, CRISPR/Cas9. This will allow scientists to better investigate parallel information processing underlying the sense of smell.

## Introduction

The complexity of the brain, in part, originates from the diversity of its components, the rich variety of cells. This diversity is evident in morphology, connectivity, molecular expression profiles, and biophysical properties (Sanes and Masland, 2015; Zeng and Sanes, 2017; Luo et al., 2018), which together give rise to what we refer to as cell types. Because the differences are thought to reflect distinct computational tasks or functions (Masland, 2004; Luo et al., 2018), the ability to selectively identify, and to manipulate, each cell type experimentally is key to understanding how the brain works.

In rodents, complex, synaptic processing of olfactory information in the brain first occurs in the olfactory bulb (OB). The principal cells of the OB, the mitral cells (MCs) and tufted cells (TCs), convey the output of this region and are thought to form parallel information streams. They differ in a variety of anatomic and physiological properties (Fukunaga et al., 2012; Igarashi et al., 2012; Phillips et al., 2012; Otazu et al., 2015; Economo et al., 2016; Kapoor et al., 2016; Jordan et al., 2018). MCs, which are the larger of the two, are thought to form distinct circuits with local neurons from those formed by TCs (Mori et al., 1983; Fukunaga et al., 2012; Phillips et al., 2012; Geramita et al., 2016), some of which may explain the differences in how they encode odors. For example, in TCs, the timing of responses adheres strictly to a specific phase of the sniff cycle, whereas MCs modulate the timing widely over the entire sniff cycle (Fukunaga et al., 2012; Igarashi et al., 2012; Ackels et al., 2020). Signal integration over this long temporal window is thought to allow MCs to represent more complex information (Fukunaga et al., 2012). Further, in contrast to TCs whose axons project to a more limited portion of the olfactory cortex, the target areas of MCs range widely, extending as far as the posterior piriform cortex, the cortical amygdala, and the lateral entorhinal cortex (Haberly and Price, 1977; Igarashi et al., 2012), indicative of a variety of behavioral contexts in which MCs are likely to be important.

Despite the fundamental roles these two cell types play in olfaction, suitable molecular markers and genetic tools are lacking. Molecules commonly used to label the output neurons of the OB include protocadherin-21 (Nagai et al., 2005), T-box transcription factor 21 (*Tbx21*, also known as *Tbet*) (Papaioannou and Silver, 1998; Faedo et al., 2002), as well as cholecystokinin (*Cck*) (Seroogy et al., 1985). In the brain, *Tbx21* is expressed from embryonic day 14 (Faedo et al., 2002) and is exclusive to the principal neurons of the OB, labeling both MCs and TCs (Faedo et al., 2002; Mitsui et al., 2011; Haddad et al., 2013). In contrast, *Cck* is expressed widely in the brain (Larsson and Rehfeld, 1979; Taniguchi et al., 2011). In the OB, expression occurs preferentially in TCs over MCs (Seroogy et al., 1985), which has been used for analyzing the unique physiology of TCs (Economo et al., 2016; Short and Wachowiak, 2019). These overlapping but differential expression patterns between *Cck* and *Tbx21* may be useful in discovering more selective markers to distinguish the two types of principal neurons.

A variety of methods now exist to analyze gene expression patterns in relation to cell types, including In-situ hybridisation (ISH) (Lein et al., 2007), as well as transcriptomic approaches that retain spatial information, with increasing resolution (Ståhl et al., 2016). More recently, single-cell RNA sequencing (scRNA-seq) (Sugino et al., 2006; Tang et al., 2009; Pfeffer et al., 2013; Zeisel et al., 2015; Shekhar et al., 2016; Tasic et al., 2016) has seen rapid developments, which have enabled the investigation of cell type-specific gene expression patterns with unprecedented levels of detail and scale (Lein et al., 2017; Zeng and Sanes, 2017). A useful application of this information in turn may be to generate transgenic driver lines that allow a particular cell type to be extensively studied. The availability of Cre-driver lines has been instrumental in revealing unique functions of distinct cell types, across multiple levels of analyses (Gong et al., 2003; Taniguchi et al., 2011; Madisen et al., 2012, 2015; Dhande et al., 2013; Cruz-Martín et al., 2014; Wolff et al., 2014; Sanes and Masland, 2015; Daigle et al., 2018).

Here, we take advantage of a large dataset that has become publicly available (Zeisel et al., 2018), to discover markers that distinguish between MCs from TCs. The results of the analyses allowed us to generate, and characterize, new Cre-driver lines. Such molecular tools will be key to understanding the mechanisms of olfactory perception and behavior.

## Materials and Methods

### 

#### Gene expression data

scRNA-seq data from the mouse brain were obtained from Zeisel et al. (2018) in Loom format. We used the dataset from the level 2 analysis that corresponds to the olfactory neurons. The gene expression table represents the expression levels of 27,998 genes in 10,745 OB cells. The gene expression level, which counts the number expressed, was transformed into log2(count + 1) before analyzing further.

##### Dimensionality reduction

**Figure 1.**
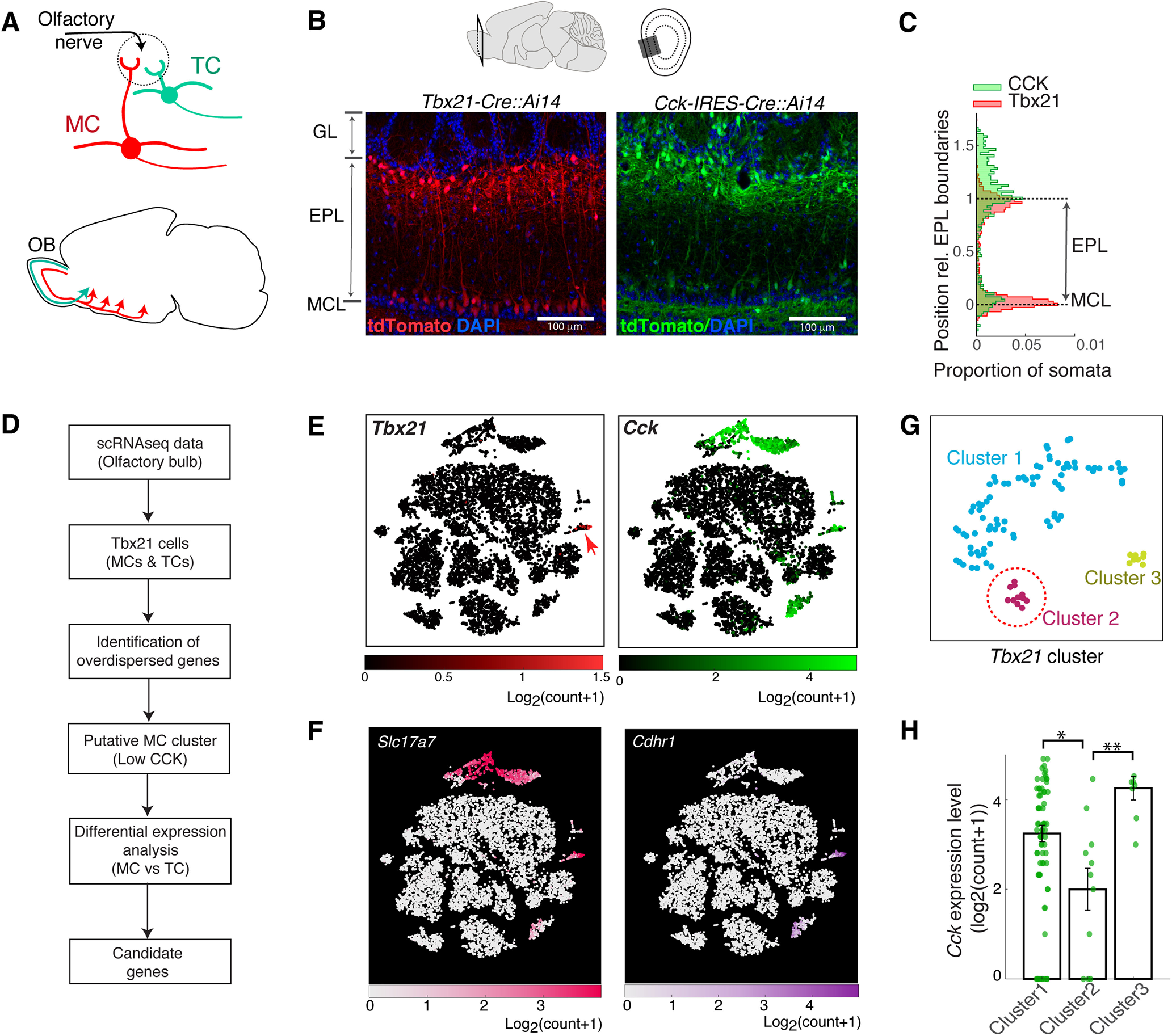
Strategy for identifying mitral-cell specific markers from scRNA-seq data. ***A***, Schematic showing major anatomic differences between the two cell types; MCs (red) are located deeper in the OB layers and project widely in olfactory cortices. TCs (green) are smaller, superficially located principal neurons that project to anterior portions of the olfactory cortex. ***B***, *Tbx21* and *Cck* expression patterns in the main OB; example images showing tdTomato expression patterns in *Tbx21*-Cre::Ai14 mouse (red) and *Cck-IRES-Cre::Ai14* mouse (green). Scale bar, 100 µm. ***C***, Soma positions of tdTomato-expressing cells relative to the EPL boundaries, for the images shown in ***B***. EPL depth was normalized so that it ranged from 0 to 1, with the lower boundary (MCL) corresponding to 0. ***D***, Schematic of workflow; putative mitral cluster from scRNA-seq data is identified by the observation that MCs and TCs both express *Tbx21*, but *Cck* is more abundant among TCs. Once putative MC and TC clusters were identified, differential expression analysis was conducted to identify genes that are selectively expressed in MCs. ***E***, OB cells plotted in tSNE coordinates, with *Tbx21* and *Cck* expression levels (left and right, respectively) indicated with color maps shown below. ***F***, Expression levels of common markers for projection neurons of the OB; VGlut1 (*Slc17a7*) and *Cdhr1*. ***G***, *Tbx21*-positive cluster was further analyzed and the subclustered and displayed in new tSNE coordinates. ***H***, *Cck* expression levels for the subclusters in ***E***. Cluster 2 has the lowest level and is inferred to be the putative MC cluster (red dotted line in ***E***). Statistical significance: *p = 0.05; **p = 0.01. For details, see Experimental design and statistical analysis.

We screened for genes that have higher variability than expected by calculating a log-transformed Fano factor for each gene, as previously described (Li et al., 2017):

$$F\left(x\right)={log}_{10}\left[\frac{\sigma^{2}\left(x\right)}{µ\left(x\right)}\right],$$

where µ$\left(x\right)$ and $\sigma^{2}\left(x\right)$ are the mean and the variance of the expression level across cells, respectively. Then using the mean expression across different cells, we split the genes into 20 subsets and calculate the *Z* score of the Fano factor within each subset as follows:

$$Z\left(x\right)=\frac{\left[F\left(x\right)-mean\left(F\left(x\right)\right)\right]}{std\left[F\left(x\right)\right]},$$

where $mean\left(F\left(x\right)\right)$ and $std\left[F\left(x\right)\right]$ are the mean and the SD of $F\left(x\right)$ within the subset. The top 500 genes with the highest value of $Z\left(x\right)$ were used to cluster the gene expression data. To visualize and cluster the gene expression data corresponding to individual cells in 2D space, we reduce the dimensionality using the principal component analysis (PCA) and *t*-distributed Stochastic Neighbor Embedding (tSNE) (van der Maaten and Hinton, 2008). We used top 10 principal components to run tSNE with the following parameters: learning rate = 10, perplexity = 33. The result is shown in Figure 1. Since many genes have similar expression patterns across different cells, to increase the power of PCA and tSNE, we extracted overdispersed genes (i.e., the most informative genes).

##### Clustering

Using the two-dimensional data above, hierarchical clustering algorithm HDBSCAN (Campello et al., 2013) was performed with the following parameters: min_clust_size = 5, min_pts = 13, which indicates which nearest neighbor to use for calculating the core distances of each point. The cluster with the highest expression level of Tbx21 gene contained 101 cells. Within this group, clustering with HDBSCAN on tSNE space revealed four clusters. We then compared the *Cck* expression level across the clusters. We found that 18% (2 of 11) of cells from Cluster 4 express *Cck* above the threshold = 3 (log2(count + 1), while 64% (58 of 90) from Clusters 1, 2, and 3 express *Cck* above the threshold; thus, we defined the Cluster 4 to be the putative MC cluster.

##### Differential expression analysis and identification of molecular markers

We used the Mann–Whitney *U* test to find differently expressed genes. The test works under the assumption that the samples are independent. *p* values were adjusted using the Benjamini–Hochberg procedure. We screened for significant genes, with the adjusted *p* value below the threshold = 0.05, where its median expression level in the MC cluster above the threshold = 3 in >50% of the cells. Of these, genes that are highly expressed in non-MC clusters were eliminated (cutoff of 10% of cells).

#### Animals

All animal experiments were approved by the animal experiment ethics committee of Okinawa Institute of Science and Technology Graduate University (protocol: 2018-201) and University of Tsukuba. ICR and C56BL/6J mice were purchased from Laboratories International for generation of transgenic mice, and C56BL/6J from Japan CLEA for subsequent breeding. *Ai14* (Madisen et al., 2010) were from The Jackson Laboratory, and *Ra13-Cre* was from the GENSAT project (Gong et al., 2003), via MMRRC (MBP, University of California, Davis). Mice of either sex were used in this study.

##### Generation of *Pkib-IRES-cre* and *Lbhd2-IRES-CreERT2* mice

Vector construction for knock-in mouse production was as follows: The CRISPR target sequence (5′-ATAGCAGCTATGTATTCCTGGGG-3′) was selected for integration of the IRES-Cre sequence just after the stop codon of *Pkib* and *Lbhd2*. The *pX330* plasmid, carrying both gRNA and Cas9 expression units, was a gift from Feng Zhang (Addgene plasmid 42 230) (Cong et al., 2013). The oligo DNAs (Pkib-CRISPR F: 5′-caccATAGCAGCTATGTATTCCTG-3′, and Pkib-CRISPR R: 5′-aaacCAGGAATACATAGCTGCTAT-3′) were annealed and inserted into the entry site of *pX330* as described previously (Mizuno et al., 2014). This plasmid was designated as *pX330- Pkib*. The donor plasmid *pIRES-Cre-Pkib* contained the IRES sequence (Bochkov and Palmenberg, 2006), nuclear translocation signal-Cre, and rabbit globin polyadenylation signal sequence. The 1.6 kb 5′-arm (from 1521 bp upstream to 64 bp downstream of *Pkib* stop codon) and the 2.0 kb 3′-arm (from 65 bp downstream to 2038 bp downstream of *Pkib* stop codon) were cloned into this vector. DNA vectors (*pX330-Pkib* and *pIRES-Cre-Pkib*) were isolated with a FastGene Plasmid mini Kit (Nippon Genetics) and filtrated by MILLEX-GV 0.22 µm Filter unit (Merck Millipore) for microinjection. Mice were kept in IVC cages under specific pathogen-free conditions in a room maintained at 23.5 ± 2.5°C and 52.5 ± 12.5% relative humidity under a 14 h light:10 h dark cycle. Mice had free access to commercial chow (MF diet; Oriental Yeast) and filtered water.

##### Microinjection and genomic DNA analyses

The pregnant mare serum gonadotropin (5 units) and the human chorionic gonadotropin (5 units) were intraperitoneally injected into female C57BL/6J mice with a 48 h interval and mated with male C57BL/6J mice. We collected zygotes from oviducts in mated females, and a mixture of the *pX330- Pkib* (circular, 5 ng/µl) and *pIRES-Cre-Pkib* (circular, 10 ng/µl) plasmids was microinjected into 148 zygotes. Subsequently, surviving 137 injected zygotes were transferred into oviducts in pseudopregnant ICR females and 21 pups were obtained.

To confirm the knock-in mutation, the genomic DNA was purified from the tail samples using the PI-200 DNA extraction kit (Kurabo Industries) according to manufacturer's protocol. Genomic PCR was performed with KOD-Fx (Toyobo). The primers (Cre forward: 5′-TCTGAGCATACCTGGAAAATGCTTCTGT-3′, and Pkib reverse: 5′-GTACCAGGAGCTCAAGACAACCTTACCC-3′) were used for checking the 5′ side correct knock-in, and the primers (Pkib forward: 5′-CTATTTCACAGGTCCAGTTGCTGAAACC-3′, and Cre reverse: 5′-ACAGAAGCATTTTCCAGGTATGCTCAGA-3′) were used for checking the 3′ side correct knock-in. We found that 5 of 21 founders carried the designed knock-in mutation. In addition, we checked random integration of *pX330-Pkib* and *pIRES-Cre-Pkib* by PCR with ampicillin resistance gene detecting primer (Amp detection forward: 5′-TTGCCGGGAAGCTAGAGTAA-3′, and Amp detection reverse: 5′-TTTGCCTTCCTGTTTTTGCT-3′) and no founder carried the random integration allele.

#### Histology

##### ISH

ISH was performed using the RNAscope ISH system (ACDBio) (Wang et al., 2012).

##### Brain extraction

Whole brains were extracted and immediately placed in 4% PFA, dissolved in PB (225.7 mm NaH_2_PO_4_, 774.0 mm Na_2_HPO_4._ pH 7.4) at 4°C for 24 h. Subsequently, the tissues were sunk in DEPC-treated 30% sucrose solution (∼2 d), then embedded in OCT (4583, Sakura Finetech) in a cryomold (Peel-A-Way, Sigma Millipore) to be frozen in an ethanol/dry ice bath and stored at −80°C until use.

##### Probe design

ISH was conducted using RNAscope (ACDBio), and probes were produced by ACDBio to be compatible for the procedure. Sequence regions for the *Pkib* and *Ldhd2* probes were selected using the NCBI genetic database. For both probes, regions that were common to all splice variants of each gene were selected. The *Pkib* probe targeted the region 141-973 bp of the transcript XM_006512605.3. The *Ldhd2* probe targeted the region 138-715 bp of the transcript XM_006516048.1. The *Tbx21* probe, which targets the region 893-2093 bp of the transcript NM_019507.2, was already commercially available (403331, ACDBio).

##### Hybridization

On the day of ISH, coronal OB sections (20 µm) were cut on a cryostat (Leica CM3050S, Leica Biosystems) at −20°C, washed in RNase-free PBS (Corning), and immediately mounted on glass slides (Superfrost plus, Thermo Fisher Scientific). Slides were dried for 30 min at 60°C and postfixed for 15 min in 4% PFA at 4°C. Slides then underwent ISH using RNAscope reagents, according to the manufacturer's protocols. Unless otherwise stated, all reagents were provided in the RNAscope kit (RNAScope Intro Pack 2.5 HD Reagent Kit Brown-Mm, catalog #322371). Briefly, slides were dehydrated through an ethanol series (75%, 90%, 100%, 100%, Sigma Millipore) and endogenous peroxidase activity blocked using provided hydrogen peroxide for 10 min at room temperature. Sections then underwent antigen retrieval by submersion into boiling (∼98°C-102°C) 1× Target Retrieval Solution for 5 min and were rinsed in distilled water by submerging 5 times. Subsequently, slides were submerged into 100% ethanol 5 times and air dried. A barrier using an ImmEdge hydrophobic barrier pen was drawn around the sections and left overnight at room temperature to dry. On the following day, slides were treated with Protease Plus and incubated in an oven (HybEZ II System, ACDBio) for 30 min, followed by a series of incubations in the same oven with provided solutions (AMP1-AMP6) to amplify probes (AMP1 and AMP3: 30 min at 40°C; AMP2 and AMP4: 15 min at 40°C; AMP5: 30 min at room temperature; AMP6: 15 min at room temperature). After amplification, a DAB reaction was conducted (1:1 mixture of DAB-A and DAB-B solutions, Vector Labs) for 10 min at room temperature. Slides were washed by submersion 5 times in 2 changes of distilled water.

##### Counterstaining

OB sections were immersed in Mayer's hematoxylin solution (MHS16, Sigma Millipore) for 10 min. Excess stain was washed in distilled water, and sections were differentiated by quick submersion in 0.2% ammonium hydroxide in distilled water, followed by washing for 5 min in distilled water. Slides were then dehydrated through a series of ethanol for 5 min each, followed by two 5 min immersions in xylene. Slides were then covered with DPX mountant (06522, Sigma Millipore) for histology and left at room temperature to dry before imaging.

#### Virus injection

Three-week-old, heterozygous *Lbhd2-IRES-CreERT2* mice were anesthetized with isoflurane (IsoFlo, Zoetis Japan) and placed on a stereotaxic frame (Kopf). Carprofen (Rimadyl, Zoetis; s.c., 5 mg/kg in saline) was injected subcutaneously for analgesia. The fur was trimmed, and the skin was disinfected with 10% iodine solution before incision. A craniotomy was made bilaterally over center of the dorsal OB (coordinates relative to bregma: AP: 4.8 mm; ML: ± 0.8 mm); 100 nl of AAV1-pCAG-Flex-EGFP-WPRE (Addgene) was injected from a pulled glass capillary tube (tip diameter ∼10 µm) at a depth 0.3 mm relative to the brain surface, at a rate of 2 nl every 5 s, using a Nanoject III injector (Drummond Scientific). Following injection, the glass capillary was left in place for 1 min and then slowly withdrawn. The surgical site was then sutured, and mice allowed to recover in a warmed chamber until fully awake, before being returned to their home cage. It is advisable that AAVs for conditional expression are tested before use, as they can exhibit off-target, “leak” expressions depending on the production protocol (Fischer et al., 2019), especially if not diluted enough.

#### Tamoxifen administration

Tamoxifen solution was dissolved at a concentration of 8 mg/ml in a solvent consisting of 5% ethanol and 95% corn oil (23-0320, Sigma Millipore), for once daily injections of 80 mg/kg (10 ml/kg injection volume). Tamoxifen powder (T5648, Sigma Millipore) was initially suspended in 99.7% ethanol and mixed using a Vortex mixer to allow partial dissolution. Corn oil was subsequently added to make up solution to the final volume, and the solution was heated up to 60°C with agitation on an orbital mixer in an oven, with periodic mixing on the Vortex mixer. When fully dissolved (∼30 min), the solution was cooled to room temperature, and mice were injected intraperitoneally using a 26G needle with care taken to avoid bubble formations. Injected mice were housed separately from untreated littermates. The mouse weights were monitored carefully throughout the injection period as well as 3 d after the final injection to ensure recovery. For a proof-of-principle P7 injection, 1 injection of 80 mg/kg was given using a 30G needle. A single-dose protocol was used to minimize disturbance to the pups and the nursing mother. Gloves were rubbed with the cage bedding before handling, and injected pups were returned to the cage with the mother.

#### Two-photon functional imaging

Cal-520 dextran (MW ∼11,000, AAT Bioquest) was dissolved to 50 mg/ml in Ringer's solution comprising the following (in mm): NaCl (135), KCl (5.4), HEPES (5), MgCl_2_ (1), CaCl_2_ (1.8). Cal-520 dextran solution was electroporated in the glomerular layer (GL) of the left OB of P42 *Lbhd2-CreERT2::Ai14* mice (tamoxifen dose = 3× 80 mg/kg starting at P21), at a depth ∼100 µm below the brain surface, under isoflurane anesthesia. Parameters of electroporation were set according to the low-intensity protocol described by Hovis et al. (2010). Immediately after the electroporation, the craniotomy was sealed with an imaging window, and mice were anesthetized with ketamine/xylazine (100 mg.kg^−1^/20 mg.kg^−1^, i.p.) and two-photon imaging of dye-loaded TCs and MCs was obtained with a custom two-photon microscope (INSS) using 980 nm high-power laser (Insight DeepSee, SpectraPhysics) fitted with a water-immersion 25× objective (CFI75 Apo 25XC W 1300, Nikon) and resonance scanner (30 Hz frame rate; FOV was 256 × 256 µm, 512 × 512 pixels). MCs were those located ∼ 300 µm below the brain surface (labeled represents red fluorescent cells + green fluorescence; unlabeled represents loaded cells without red fluorescence), while TCs were smaller cells located more superficially. Strongly fluorescent cells were excluded from analysis. Five odors were presented in a randomized order using a custom-made, flow-dilution olfactometer (Koldaeva et al., 2019), at ∼5% of the saturated vapor, while the total flow rate was 2 L/min. Odors used were ethyl butyrate, methyl tiglate, methyl butyrate, acetophenone, and methyl salicylate. Intertrial interval was 30 s during which lines were purged with pressurized air to minimize cross contamination. Because of bleaching and other time-dependent factors, such as the depth of anesthesia, typically, 3 or 4 presentations were given for each odor. For GCaMP6f imaging, *Lbhd2-CreERT2::Ai95D* mice were injected with tamoxifen intraperitoneally at P21 (1× 160 mg/kg). After 2 weeks, the mice were surgically implanted with a cranial window over the left OB, as well as a head plate, and allowed to recover. After 2 further weeks, they were anesthetized with ketamine/xylazine, and head-fixed for imaging. Odors were presented in the manner described above for the electroporation experiment, but 6 odors were presented with the order randomly permuted. The additional odor was butyl acetate. The body temperatures of the mice were maintained at 36°C using a thermostat.

#### Confocal imaging

Confocal images were acquired on a Zeiss LSM780 confocal microscope with a 10× objective (Carl Zeiss, NA 0.45 Plan-Apochromat) for the whole-brain sagittal sections, and 20× objective (Carl Zeiss, NA 0.8 Plan-Apochromat) for the OB. Using ZEN 2.3 software (Carl Zeiss), images were taken at a resolution of 1024 × 1024 pixels for an FOV of 850.19 µm × 850.19 µm (10×) or 425.1 µm × 425.1 µm (20× objective). To enable comparison and quantification of viral injections, imaging conditions (resolution, gain, laser power, number of averages) were kept consistent. Sequential laser excitation was used to prevent fluorophore bleed-through. Images were taken throughout the whole rostro-caudal extent of viral spread using the 20× objective. For axonal projection analysis, images were acquired using a Leica SP8 confocal microscope using a 10× (Leica Biosystems, NA 0.40 Plan-Apochromat) and a 40× (Leica Biosystems, NA 1.3 Plan-Apochromat) objective. Images were taken at a resolution of 1024 × 1024 pixels per FOV (10×: 1163.64 × 1163.64 µm; 40×: 290.91 × 290.91 µm) at sequential excitation to prevent fluorophore bleed-trough.

#### Image analysis

##### ISH signal

Images of DAB- and hematoxylin-stained OB sections were obtained using a wide-field microscope with a 10× objective in RGB, so that the hematoxylin signal could be separated into the blue channel. The same acquisition settings were used for all sections (*Tbx21*, *Lbhd2*, and *Pkib* signals). Dorsal, ventral, medial, and lateral portions of the OB at three anterior-posterior locations were imaged so that all layers (nerve layer, GL, external plexiform layer [EPL], MC layer [MCL], and GCL) were captured. To extract the positions of the EPL boundaries, in ImageJ, a binary mask from the hematoxylin signal (blue) was obtained by setting a threshold and summed along the axis parallel to the OB layers. Hybridization signal (DAB; red channel) was converted into the binary mask, also by setting a single threshold across all conditions. Pixel coordinates were normalized such that the boundaries of EPL were set from 0 to 1, with MCL being 0. The density of the hybridization signal was obtained by averaging the binary signal along the axis parallel to the OB layers.

##### Soma detection and quantification for OB

Images (1024 × 1024 pixels corresponding to 425.1 µm × 425.1 µm) taken with a 20× objective were sampled at anterior, dorsal, and ventral locations of the mid-sagittal plane for the tdTomato signal using the *Ai14* reporter line, using the DAPI channel to guide sampling, and 10 consecutive planes at a 100 µm interval for the virus injection experiment. Using only the red and green channel for tdTomato labeling and EGFP labeling, respectively, somata were detected manually in ImageJ using the ROI manager and their coordinates exported into MATLAB, without the observer knowing the identity of the mouse. EPL boundaries were demarcated using only the DAPI signals from images, using a custom-written MATLAB routine and the boundary coordinates were stored. The soma depths from above were normalized along the EPL using the boundary coordinates, such that the MCL was defined as 0, and the lower boundary of the GL as 1. One-way ANOVA was used to compare the means, using the *anova1* function in MATLAB, and the *multcompare* function with the crucial value tested with Tukey's honest significant difference criterion for *post hoc* multiple comparisons. Cells belonging to MCL were defined as those whose somata are positioned within 30% of the normalized EPL boundary from the MCL. This corresponded, on average, to 43.6 µm, which is equivalent to the lengths of two MC somata (Nagayama et al., 2010). Thus, our measure takes into consideration the displaced MCs.

##### Dendrite detection and quantification

Images used were the same as those used to detect somata above. To emphasize signals originating from dendrites, which are thin processes, background signal was subtracted from the green or red channel using *Subtract Background* function in ImageJ, with the rolling ball radius set to 5 pixels. Binary masks were created with a single threshold value, and the presence of the signal along the normalized EPL depth at each lateral position was averaged to obtain the density. The dendritic preference index was used to compare the dendritic signal in the upper EPL versus lower EPL, as a proportion of the total dendritic signal detected, calculated as follows:

$$({\mathrm{Signal\_density}}_{\mathrm{upper\_EPL}}-{\mathrm{Signal\_density}}_{\mathrm{lower\_EPL}})/({\mathrm{Signal\_density}}_{\mathrm{upper\_EPL}}+{\mathrm{Signal\_density}}_{\mathrm{lower\_EPL}})$$

##### Analysis of labeled MCs on a standardized coordinate

Labeled MCs from coronal sections (1024 × 1024 pixels, 1.2 µm per pixel) were automatically detected in ImageJ by converting the red fluorescence image into binary masks by thresholding and converted into ROIs using the Analyze Particles function (100-600 pixels, circularity 0.1-1). The MCL was delineated using the DAPI channel in MATLAB using the drawpolygon function. The line was interpolated, and labeled MCs were projected on the MCL coordinates. The center of the OB was calculated as the center of the MCL coordinates. To pool data across mice, MCL coordinates were standardized such that it ran from 0 to 2 π radians relative to the center of the OB.

##### Whole-brain somata detection

Positions of somata labeled with tdTomato were automatically detected in the red channel of the stitched confocal images. To automatically detect the labeled somata, background fluorescence was subtracted using ImageJ's *Subtract Background* function (100 pixels), then further sharpened to accentuate the somata locally using ImageJ's *Unsharp* filter with the radius set to 14 pixels, and mask weight set to 0.6. Then a binary mask was obtained by setting a threshold and the *Analyze Particles* function was used to detect round objects (size = 70-600 pixels, circularity 0.1-1), and detected structures added to the ROI manager, and exported as a list. Using the DAPI signals in the blue channel, boundaries of each nucleus were manually drawn in MATLAB using the *drawpolygon* function. Finally, for each anatomic region, all detected soma positions within the boundary were counted using the *inROI* function and normalized by the area to standardize the density of detected cells per mm^2^. Distributions of labeled somata across strains were tested with two-way ANOVA using MATLAB's *anovan* function.

#### Experimental design and statistical analysis

The Mann–Whitney *U* test, *t* test, Kolmogorov–Smirnov test, and one-way- and two-way-ANOVA were conducted using MATLAB. Unless otherwise stated, *t* tests were performed unpaired. Paired tests are described as “two-sample *t* test” in the text. For *post hoc* comparisons following significant ANOVA tests, the *p* values are given in Table 1 for brevity of figure legends. Only the significant comparisons are listed because of the large number of pairwise comparisons.

**Table 1.** Details of *post hoc* pairwise statistical comparisons

Figure	Test used	*p*
1*H*	One-way ANOVA followed by Tukey-Kramer multiple comparisons	*p* = 0.045 and *p* = 0.0086 for Cluster 1 vs Cluster 2 and Cluster 2 vs Cluster 3, respectively, for the null hypothesis that the two means do not differ.
2*G*	One-way ANOVA followed by Tukey-Kramer multiple comparisons	*p* = 0.0008 for *Tbx21* vs *Lbhd2*; *p* = 0.0035 for *Tbx21* vs *Pkib,* respectively, for the null hypothesis that the two means do not differ.
7*E*, left	One-way ANOVA followed by Tukey-Kramer multiple comparisons	*p* = 0.012 for *Tbx21* vs *Lbhd2* (1 × 80 mg/kg tamoxifen); 0.045 for *Tbx21* vs *Lbhd2* (3 × 80 mg/kg tamoxifen); 0.008 for *Ra13* vs *Lbhd2* (1 × 80 mg/kg tamoxifen); *p* = 0.029 for Ra13 vs Lbhd2 (3 × 80 mg/kg tamoxifen), each for the null hypothesis that the two means do not differ.
7*E*, middle	One-way ANOVA followed by Tukey-Kramer multiple comparisons	*p* = 8.8 × 10^−5^ for *Tbx21* vs *Lbhd2* (1 × 80 mg/kg tamoxifen); 1.5 × 10^−5^ for *Tbx21* vs *Lbhd2* (3 × 80 mg/kg tamoxifen); 6.5 × 10^−5^ for *Ra13* vs *Lbhd2* (1 × 80 mg/kg tamoxifen); 1.2 × 10^−5^ for *Ra13* vs *Lbhd2* (3 × 80 mg/kg tamoxifen), each for the null hypothesis that the two means do not differ.
7*E*, right	One-way ANOVA followed by Tukey-Kramer multiple comparisons	*p* = 3.4 × 10^−5^ for *Tbx21* vs *Lbhd2* (1 × 80 mg/kg tamoxifen); 1.1 × 10^−4^ for *Tbx21* vs *Lbhd2* (3 × 80 mg/kg tamoxifen); 2.3 × 10^−4^ for *Ra13* vs *Lbhd2* (1 × 80 mg/kg tamoxifen); 8.0 × 10^−4^ for *Ra13* vs *Lbhd2* (3 × 80 mg/kg tamoxifen), each for the null hypothesis that the two means do not differ.

Data will be available on request. *Lbhd2-CreERT2* has been donated to The Jackson Laboratory Repository (stock #036054).

## Results

In search of molecular markers, we sought to compare the gene expression patterns of MCs and TCs. This may reveal candidate markers, which are genes that are selectively enriched in the target cell type of interest, in this case MCs, but not expressed in other cell types. This first requires a method to identify MCs and TCs in a gene expression data, and, second, distinguish their gene expression profiles from each other. Previous studies observed that *Tbx21*, a T-box type transcription factor, labels both MCs and TCs (Faedo et al., 2002; Mitsui et al., 2011; Haddad et al., 2013), while the neurotransmitter cholecystokinin (*Cck*) is more abundant in TCs (Seroogy et al., 1985; Economo et al., 2016). To verify these distributions in our hands, we crossed *Tbx21-Cre* and *Cck-IRES-Cre* lines (Taniguchi et al., 2011; Haddad et al., 2013) with the Rosa-CAG-LSL-tdTomato reporter line, *Ai14* (Madisen et al., 2012), for Cre-dependent expression of the red fluorescent protein, tdTomato. We confirm that *Tbx21*-driven expression labels cells in the MCL and the EPL where TCs are located, while *Cck*-driven expression labels a larger number of cells all over the OB (Fig. 1*A–C*), especially those that extend more superficially in the GL and sporadically in the granule cell layer. Importantly, labeling coupled to *Cck* expression is less consistent in cells that occupy the MCL. These differential expression patterns between *Tbx21* and *Cck* may be used to distinguish MCs from TCs in gene expression data (Fig. 1*B*,*C*).

Identification of molecular markers by differential expression analyses requires a robust and large dataset, especially when distinguishing similar cell types, such as in the case of MCs and TCs. We turned to a public, large-scale scRNA-seq dataset of the mouse brain (Zeisel et al., 2018). This contains data from ∼0.5 million cells, 10,745 cells of which are from the OB. We clustered the data based on the similarity of gene expression patterns. To achieve this efficiently, we identified the top 500 overdispersed genes of 27,998 genes in the dataset (see Materials and Methods; Fig. 1*D*). Such genes are highly informative for determining genetic differences among the cells. Using this reduced dataset, we performed PCA, followed by tSNE on the first 10 principal components to further reduce the dimensionality of the gene expression space to two. The combination of the two algorithms preserves both the global and local structures of the data (Kobak and Berens, 2019). To obtain clusters, hierarchical density-based special clustering algorithm (HDBSCAN) (Campello et al., 2013) was applied on the two-dimensional tSNE space to cluster the data (see Materials and Methods). Within the OB dataset, we found that 1682 cells belong to *Cck*-positive clusters, while *Tbx21-*expressing cluster comprised 101 cells. Generally, expression patterns of *Cck* and *Tbx21* together mirror those of *Slc17a7* (VGlut1) and Slc17a6 (Vglut2), indicating that they are mainly glutamatergic populations (Fig. 1*E*,*F*), with the largest portion of glutamatergic, *Cck*-positive clusters residing outside of the *Tbx21*-positive cluster. Further, a small set of *Cck*-expressing neurons did not overlap with the *Slc17a7*-positive cluster (Fig. 1*E*,*F*). To identify a putative MC cluster from the scRNA-seq data, we took advantage of the observation that MCs and TCs both express *Tbx21*, but *Cck* is more abundant among TCs. In the *Tbx21*-positive cluster, the second largest cluster (Cluster 2; Fig. 1*G*) showed the lowest *Cck* expression level (Fig. 1*H*). We thus refer to this as the putative MC cluster, and refer to the remaining as TC Cluster 1 (TC1).

**Figure 2.**
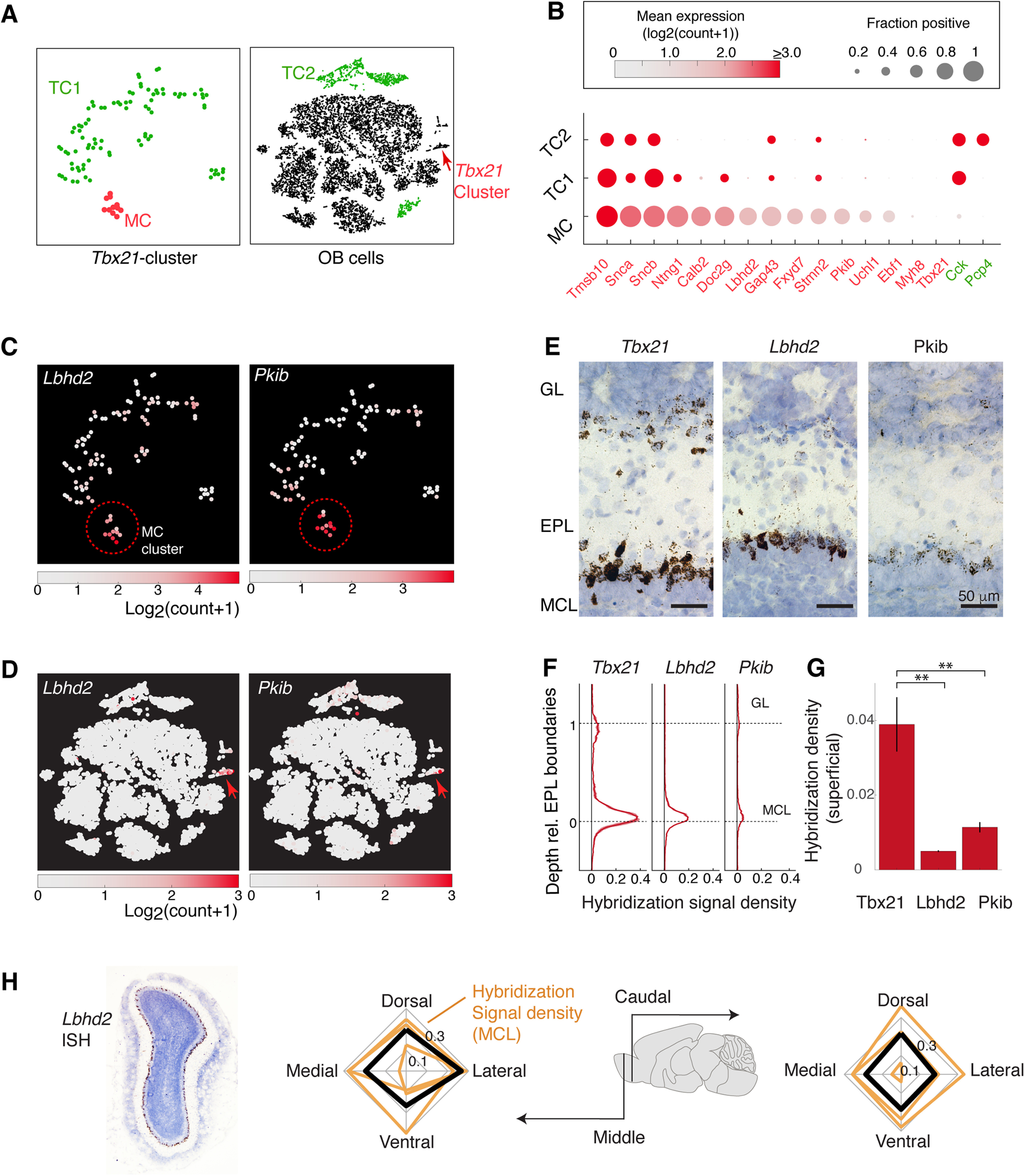
Differential gene expression analysis reveals candidate marker genes for MCs. ***A***, Cluster grouping used for differential gene expression analysis to distinguish TCs from MCs. *Tbx21*-expressing cells (left) constitute the MC cluster (red) and TC1 cluster (green), which is identical to the combined Clusters 1 and 3 shown in Figure 1*G*. The OB-wide dataset (right) contains the TC2 cluster (green), which is equivalent to the *Cck*-rich clusters shown in Figure 1*E* without the *Tbx21*-rich clusters. ***B***, Genes that are significantly enriched in MCs (red) and TC clusters (green). The size of data points indicates the consistency of expression, measured as the fraction of cells in the cluster that express the gene. Mean expression level (log2(count + 1)) is color-coded as shown in the color map above. ***C***, Expression levels of two candidate genes, *Pkib* and *Lbhd2*, with the corresponding color maps, superimposed on the three subclusters of the *Tbx21*-rich cluster (same tSNE coordinate as Fig. 1*G*), and (***D***) the whole OB data. Red arrow points to the *Tbx21* cluster. ***E***, Example ISH signals revealed by DAB staining for *Tbx21* (left), *Lbhd2* (middle), and *Pkib* (right) for the MOB layers indicated. Scale bar, 50 µm. ***F***, ISH signal density relative to the EPL boundary (0-1); hybridization signal was thresholded, and the proportion of pixels above the threshold for each normalized EPL depth was expressed as density. ***G***, Summary of hybridization signals in the superficial locations (depth upper half of EPL). *N* = 3 mice, with samples from dorsal, ventral, medial, and lateral locations at middle and caudal levels of the antero-caudal axis. ***H***, Quantification of regional variation; average hybridization signal density from the MCL (right) for dorsal, ventral, medial, and lateral samples taken from middle plane (bottom plot) and caudal plane (top plot) of the AP axis. Orange lines indicate data from individual mice. Black lines indicate the average across the 3 mice.

**Figure 3.**
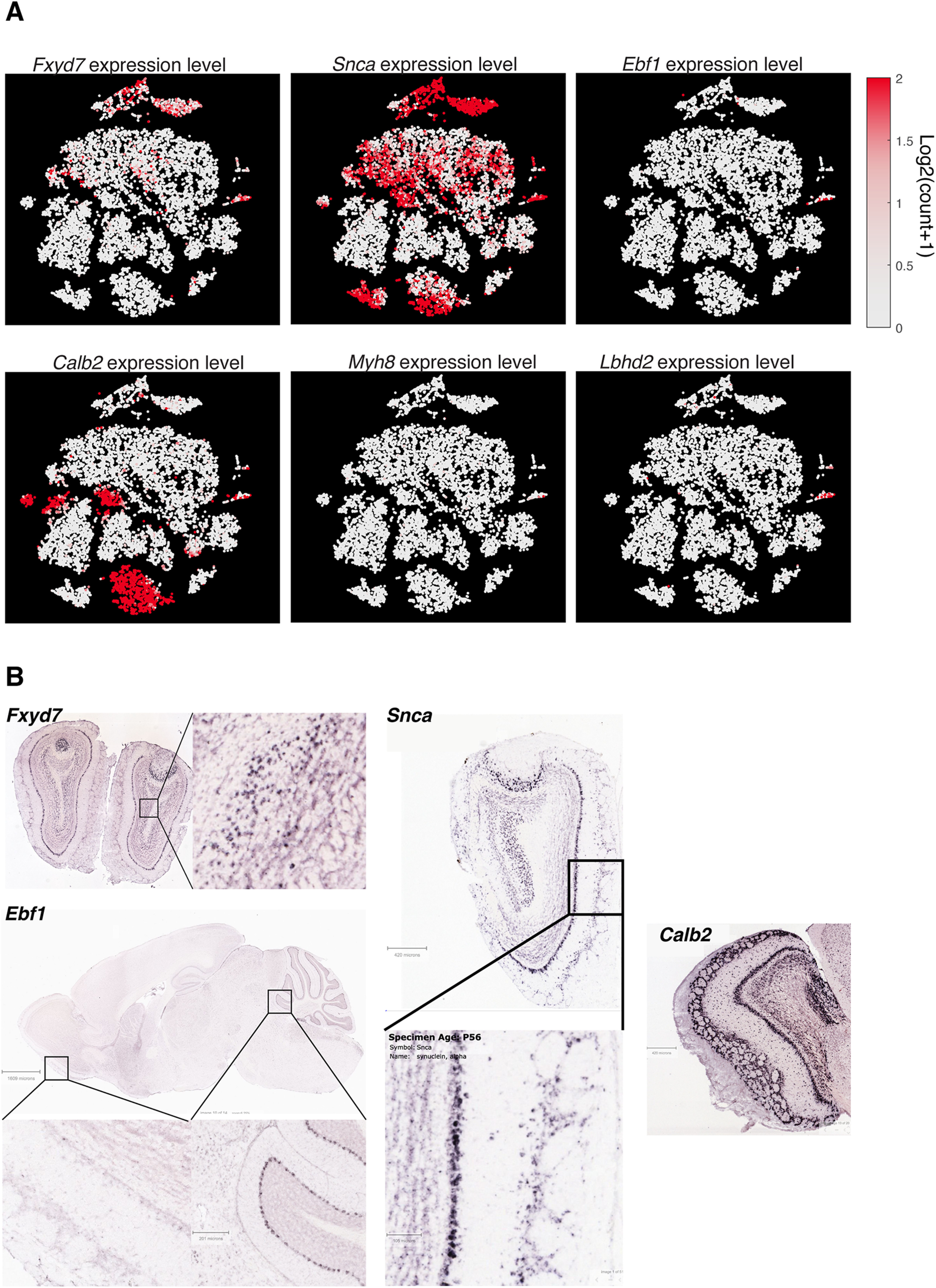
OB-wide tSNE data and Allen Brain Atlas ISH data used to screen candidate MC markers that were not analyzed further. Differential expression analysis indicates that *Fxyd7*, *Ebf1*, *Snca*, *Calb2*, and *Myh8* are significantly enriched in MCs relative to TCs. ***A***, To screen candidates, the expression pattern (color map) in the whole OB data was analyzed. The same tSNE coordinates as in Figure 1*E*, *F* are used, with *Lbhd2* expression pattern shown for comparison. ***B***, Further, the ISH database of the Allen Brain Atlas was used to assess the spatial expression patterns. *Fxyd7* seems to be expressed by neurons deep in the granule cell layer as well as superficial cells. *Snca* is expressed by some superficially located neurons as well as some neurons of the anterior olfactory nucleus. *Ebf1* is hardly detectable in the OB, although it is present in the Purkinje cell layer of the cerebellum. Dense *Calb2* hybridization signal is visible in the GL, EPL, MCL, as well as the granule cell layer. *Myh8* signal was not described in the ISH database, but it is a marker for somatostatin-positive cells in the subventricular zone (Lim et al., 2018), which is the source of SST-positive interneurons of olfactory cortices. The expression data in ***A*** show low levels of Myh8 expression in many neurons outside of the Vglut1- and 2-positive clusters. Image credit: Allen Institute.

An ideal molecular marker should be expressed abundantly and consistently in the cell type of interest, while having minimal expression levels in other cell types. To search for candidates with these properties, gene expression patterns of putative MCs were compared against the rest of *Tbx21*-expressing neurons (TC1; Fig. 2*A*), as well as glutamatergic, *Cck*-positive clusters outside of the *Tbx21*-cluster (TC2; Fig. 2*A*). First, the Mann–Whitney *U* test was used to screen genes that are differentially expressed, with *p* values adjusted using the Benjamini–Hochberg procedure. This procedure identified several differentially expressed genes (Table 2), at the adjusted *p* = 0.05 level. Among these were *Calb2* (calbindin 2), *Ntng1* (netrin G1), *Ppm1j* (protein phosphatase 1J), *Rph3a* (rabphilin 3A), *Kcnq3* (voltage-gated potassium channel subfamily Q member 3), and *Chrna2* (cholinergic receptor nicotinic α2). Of the differentially expressed genes, we focused on those that are present in the majority (>50%) of cells in the putative MC cluster, but in <10% of the cells outside of this cluster (Fig. 2*B–H*; Table 2). Only a small number of the differentially expressed genes fulfilled these criteria, and even fewer showed minimal expression levels outside of MCs, as judged by the OB-wide expression patterns (Fig. 3*A*), as well as by the ISH data in the Allen Brain Atlas (Lein et al., 2007). Candidate genes that showed clear hybridization signals outside of the MCL were therefore not pursued further (Fig. 3*B*).

**Table 2.** Genes differentially expressed between MCs and TCs

Gene name	Mean expression (log2(count + 1))	Adjusted *p*
Myh8	2.14	1.57E-09
Pkib	3.40	7.02E-06
Fxyd7	3.66	1.41E-05
A230065H16Rik (Ldbh2)	3.77	1.55E-05
Ebf1	2.55	1.55E-05
Calb2	4.60	1.55E-05
Snca	5.10	1.55E-05
C1ql1	2.53	1.55E-05
Cxcl14	0.42	1.55E-05
Sostdc1	0.36	2.07E-05
Tmsb10	6.12	3.05E-05
Spp1	2.20	3.45E-05
Ntng1	4.76	4.25E-05
RP23-407N2.2	1.81	4.70E-05
1110008P14Rik	2.45	0.000163822
Tspan17	1.99	0.000211554
Uchl1	3.24	0.000253744
Shisa3	1.98	0.000253744
Gap43	3.69	0.000439148
Ppm1j	1.85	0.000449468
Rph3a	0.74	0.000449468
Crtac1	1.97	0.000449468
Tnnc1	0.27	0.000457871
Mmp17	0.45	0.000585813
Nov	0.18	0.000707168
Gng13	1.03	0.001406527
Rab15	1.62	0.001959665
Gm27199	1.11	0.001978521
Vsnl1	0.83	0.002521565
Stmn2	3.57	0.003524909
Npr1	1.30	0.003848843
Doc2g	4.52	0.004924184
Nptxr	0.78	0.005760704
Kcnq3	0.94	0.005928465
Slc1a2	1.29	0.007685702
Sncb	4.97	0.00781742
Ephx4	1.37	0.009259688
Atp9a	1.91	0.009383075
Nrsn1	2.65	0.01005383
Diras2	1.50	0.01094975
Rgs4	0.76	0.01098611
AI413582	2.09	0.0124281
Abi3bp	0.09	0.01393499
Lingo1	1.67	0.01393499
Tshz2	3.22	0.01393499
Mal2	0.18	0.01393499
Fkbp1b	0.00	0.01393499
Prkcb	1.23	0.01504482
Cdh4	1.02	0.01504482
Meg3	7.01	0.01660475
Chrna3	0.61	0.01660475
Ifitm10	0.74	0.01905793
Adk	0.34	0.02020242
Stx1a	0.89	0.02020242
Igfbp5	2.75	0.02381658
Fam19a1	0.09	0.0261028
Grin1os	0.09	0.0261028
Cdc20	0.09	0.0261028
Gm26803	0.09	0.0261028
Resp18	1.14	0.0261028
Pantr1	1.12	0.02678842
Lmo4	0.91	0.02690157
Pvrl1	1.07	0.03585761
Kitl	0.00	0.0397583

**Figure 4.**
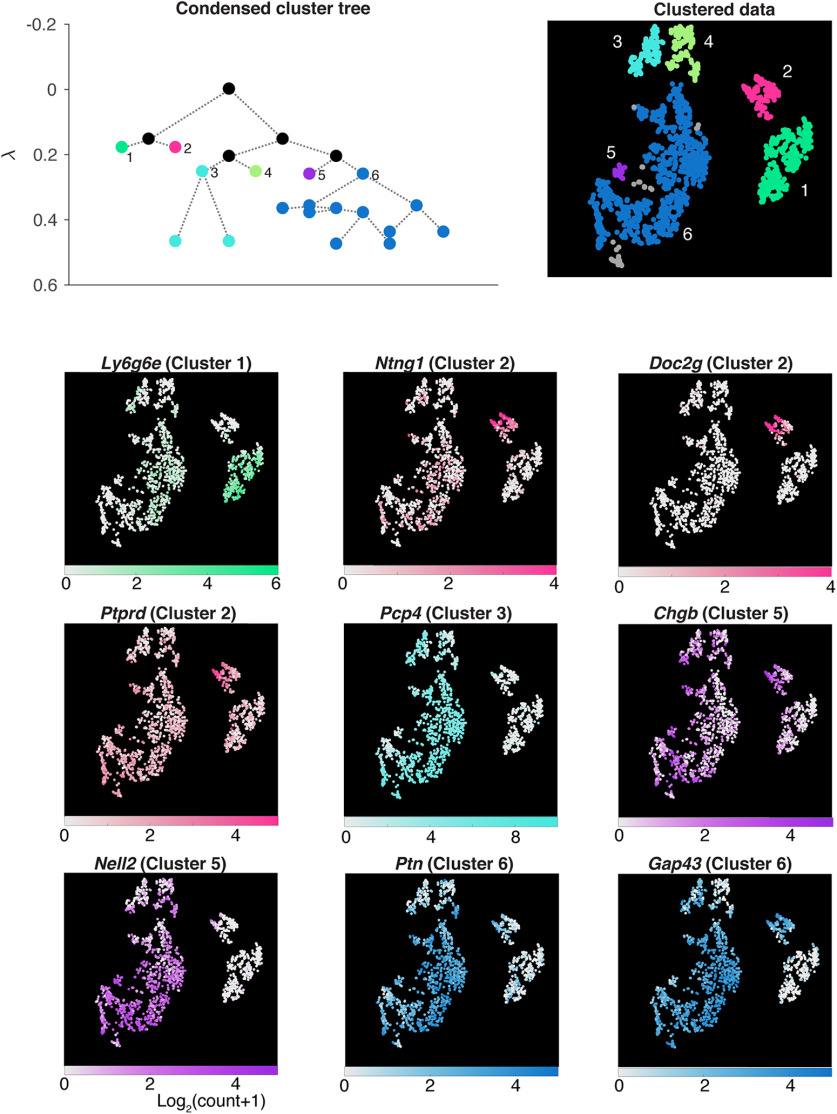
Subclustering analysis of the *Cck*-expressing cluster. The *Cck*-expressing population from the OB dataset was further analyzed to reveal subclusters. For each subcluster, candidate marker genes were identified by differential gene expression analysis, where expression patterns from a cluster of interest were compared against all other clusters combined. The expression patterns for each candidate marker, and in which subcluster the gene is enriched (in brackets), are shown for all cells in the *Cck*-positive population, with corresponding color maps. While the *Doc2g* gene selectively labels the sub-Cluster 2 in the TC dataset, it is a gene that is also abundantly expressed by MCs (see Fig. 2).

Based on the initial screening, *Pkib* (protein kinase inhibitor β) and *Lbhd2* (LBH domain containing 2; Fig. 2*C*,*D*) genes fit the criteria for an MC marker. To confirm that these genes indeed are selectively expressed in MCs, we conducted ISH for *Pkib* and *Lbhd2* (Fig. 2*B–D*) on OB sections. Indeed, probes for *Pkib* and *Lbhd2* gave rise to monolayer-like signals at the lower boundary of the EPL, corresponding to the location of the MCL. For quantification, *Pkib* and *Lbhd2* signals were expressed as density (see Materials and Methods) and plotted relative to the boundaries of the EPL. This revealed that *Pkib* and *Lbhd2* both label cells in the MCL, with significant reduction in the superficial signals corresponding to TCs, especially compared with *Tbx21* (Fig. 2*D*; mean signal densities in the upper EPL: *Tbx21* = 0.39 ± 0.007; *Lbhd2* = 0.005± 0.0002; *Pkib* =0.01± 001, *p* = 0.0007, one-way ANOVA, *F* = 17.9, degrees of freedom = 2). Hybridization signal in the MCL was relatively uniform throughout the OB (Fig. 2*H*), while residual expression patterns of *Pkib* and *Lbhd2* in non-MC cells differed somewhat, with faint signals in the GL and EPL for *Pkib* and *Lbhd2*, respectively. Thus, *Pkib* and *Lbhd2* are promising candidates for selectively labeling MCs. On the other hand, the same analysis failed to reveal clear molecular markers for subclasses of TCs (Fig. 4).

**Figure 5.**
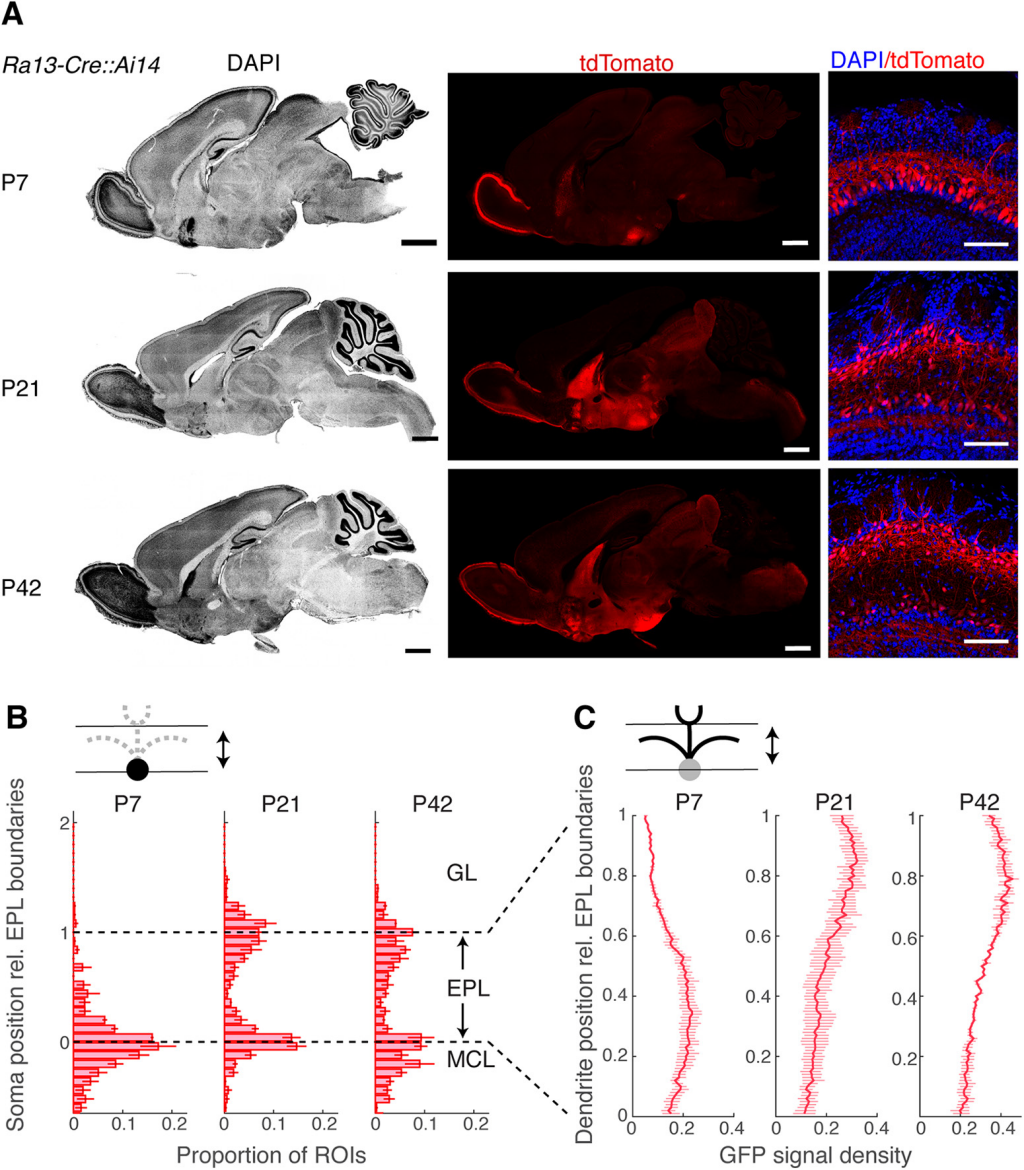
The *Ra13-Cre::Ai14* line reveals a developmental accumulation of recombination patterns outside of MCs. ***A***, Sagittal brain sections from example P7 (top row), P21 (middle row), and P42 (bottom row) mice, showing DAPI signal (left column; pseudo-colored in grayscale), corresponding tdTomato (middle column) and merged signals for OB (right column). Scale bars: whole sagittal view, 1 mm; OB, 100 µm. ***B***, Distribution of somata positions with respect to the EPL boundaries. *N* = 3 mice (average of measurements from anterior, ventral, and dorsal parts for each animal). ***C***, Distribution of labeled dendrites with respect to the EPL boundaries.

**Figure 6.**
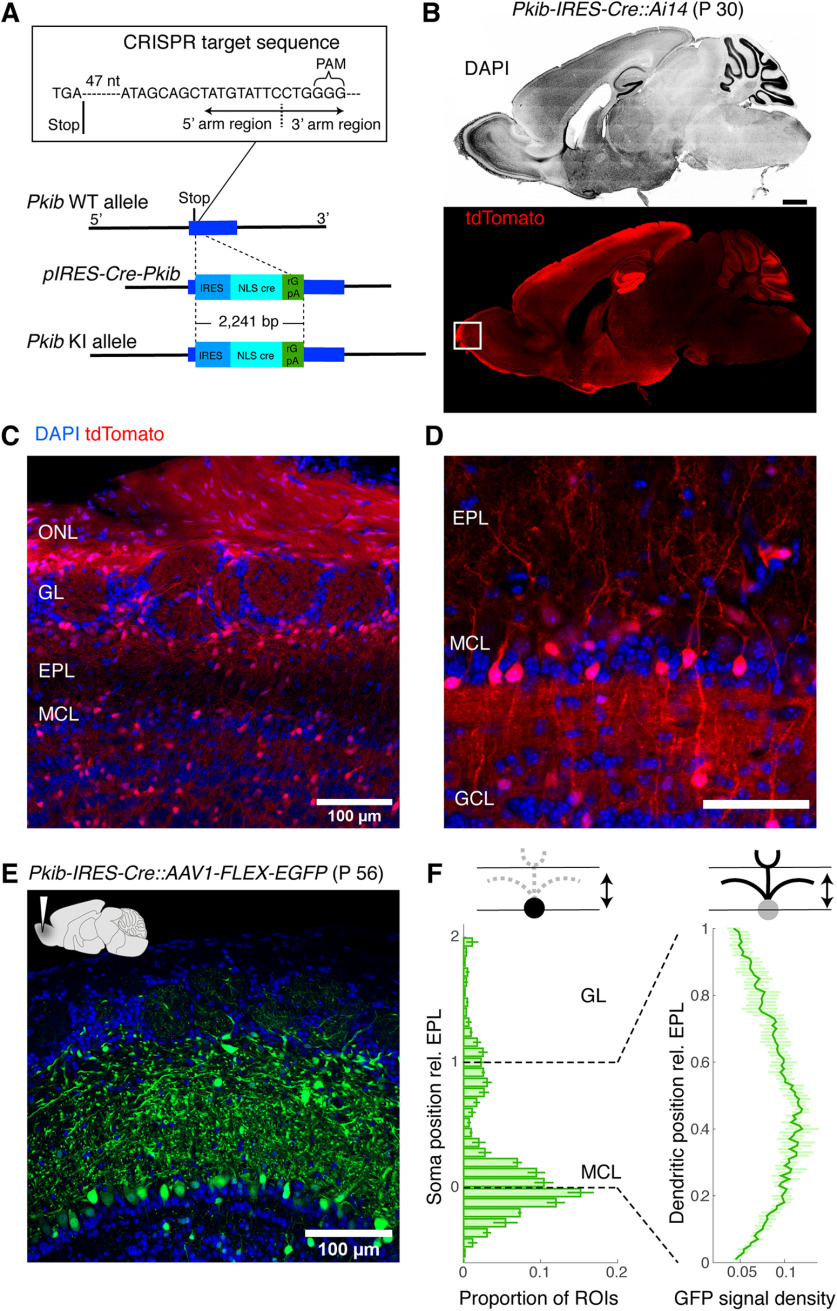
*Pkib-IRES-Cre* labels a wide variety of non-MC neurons, with late onset labeling in MCs ***A***, Strategy for CRISPR/Cas9-mediated generation of the *Pkib-IRES-Cre* transgenic mouse. The CRISPR-target sequence (inset) was just after the stop codon of the Pkib gene. Construct included sequences for IRES, Cre-recombinase with a nuclear localization signal, and rGpA. ***B***, Cre-mediated recombination pattern in a 30-d-old *Pkib-IRES-Cre::Ai14* mouse. Structures visible in this sagittal plane are revealed by DAPI (top), and the corresponding pattern of recombination revealed by tdTomato signal. Scale bar, 0.5 mm. ***C***, tdTomato expression pattern relative to the OB layers from the same animal. ONL, Olfactory nerve layer. Note the dense labeling of the ONL. ***D***, A higher magnification of image in ***C***. Signals in the MCL are faint or lacking at this developmental stage. Scale bar, 0.1 mm. ***E***, A confocal image showing the EGFP expression pattern 2 weeks after an injection of AAV1-flex-EGFP into the MOB in a 42-d-old *Pkib-IRES-Cre* mouse. ***F***, Summary of soma location (left) and dendritic signal density (right) relative to the EPL boundaries. Note the heavy presence of somata outside of the MCL and dendrites in the upper portion of the EPL.

Having identified candidate markers for MCs, we sought to test whether Cre-recombinase expression from these loci would allow MC-specific labeling. Screening several public depositories, we found a Cre-driver line for *Lbhd2* under a synonymous gene symbol (A230065H16Rik) on GENSAT, a large repository of BAC-mediated transgenic mouse lines (Heintz, 2004; Gong et al., 2007). Since the two independent Cre-driver lines (*Ra31-Cre* vs *Ra13-Cre*) show similar recombination patterns, we chose to analyze the line *Ra13-Cre*. As above, we crossed *Ra13-Cre* mice with *Ai14* reporter mice to analyze the pattern of Cre-mediated recombination in the brain (Fig. 5). At postnatal day 7 (P7), red fluorescence was highly selective, showing dense and restricted expression in the cells of the MCL of the OB (Fig. 5*A–C*; mean number of fluorescent TCs as a proportion of fluorescent cells in the MCL = 0.09 ± 0.04 for P7; *p* = 0.18, *t* test for mean = 0, t-statistic = 2; *n* = 3 mice). Correspondingly, labeled dendrites were observed preferentially in the lower portion of the EPL (fluorescence signal density = 0.20 ± 0.03 for lower EPL vs 0.10 ± 0.01 for upper EPL; *p* = 0.03, two-sample *t* test for equal means, *n* = 3 mice each), consistent with MCs having dendrites that ramify in the deeper portion of the EPL. At this developmental stage, red fluorescence was observed only sparsely in the rest of the brain, except for the lateral septum and the dorsomedial nucleus of the hypothalamus. However, in older mice, the residual recombination becomes widespread and is observed throughout the brain. In the OB at this stage, while the labeling is still restricted to the projection neurons, a substantial number of TCs also become labeled (mean number of fluorescent cells in the upper EPL as a proportion of fluorescent cells in the MCL = 1.05 ± 0.08 for P21 and 1.33 ± 0.12 for P42). A Cre-driver line that we generated for the second marker candidate, *Pkib*, was deemed unsuitable for MC-specific labeling because of late-onset expression in MCs, as well as a widespread recombination in neurons other than MCs (Fig. 6).

**Figure 7.**
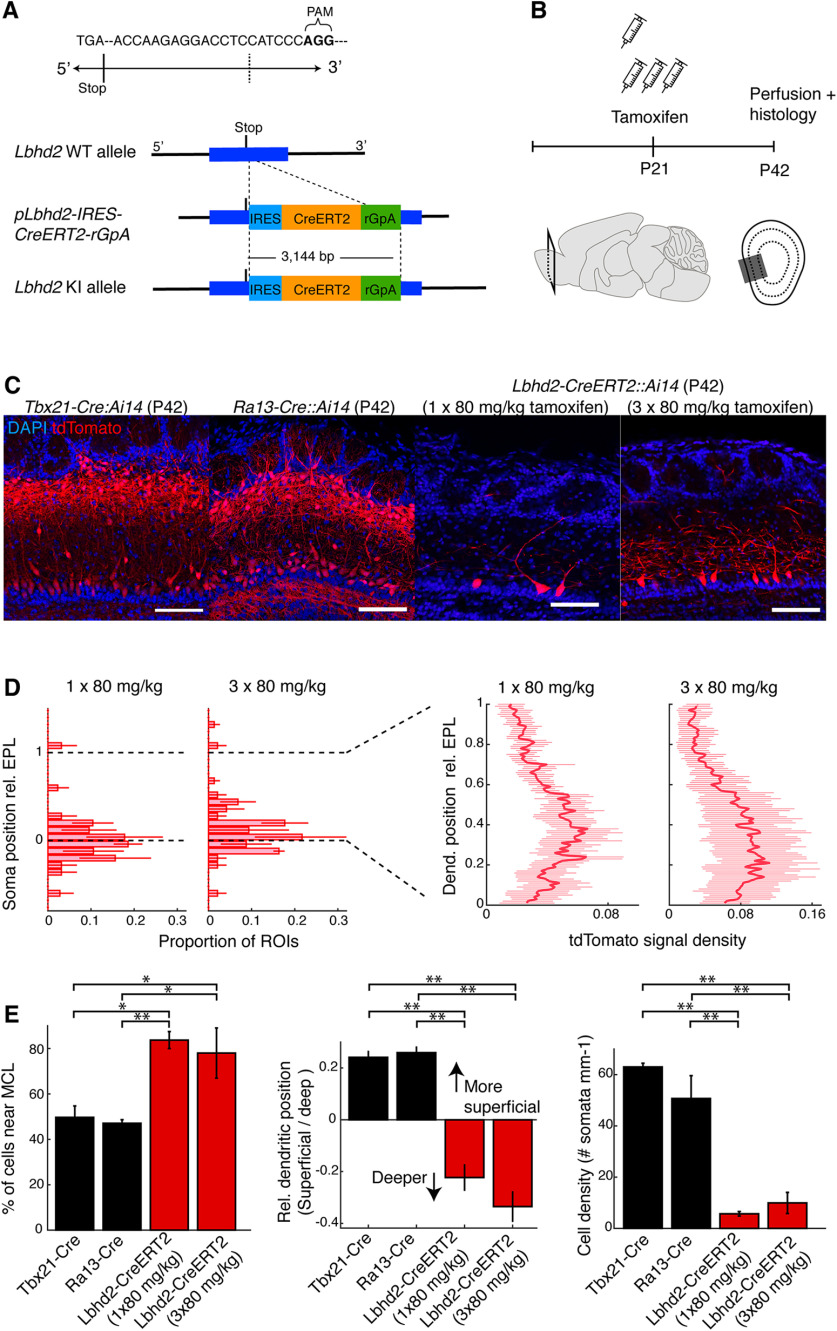
*Lbhd2-CreERT2* line achieves MC-specific labeling in the OB even in adulthood. ***A***, Constructs for CRISPR/Cas9-mediated knock-in line. IRES-CreERT2 cassette was targeted to a region immediately following the stop codon of the Lbhd2 gene. ***B***, Schematic of the tamoxifen injection protocol: tamoxifen was injected intraperitoneally at P21, and the recombination pattern in the OB was examined 3 weeks later. Cohorts of mice received injection for either 1 or 3 d at 80 mg/kg per day. ***C***, Example recombination patterns. From left: *Tbx21-Cre::Ai14*, *Ra13-Cre::Ai14*, *Lbhd2-CreERT2::Ai14* (one injection) and *Lbhd2-CreERT2::Ai14* (three injections). Scale bar, 100 µm. ***D***, Summary of labeled structures at P42 for the corresponding mouse lines, showing the proportion of cells in the MCL relative to all labeled cells (left), comparison of labeled dendrites in the superficial versus deep portions of the EPL (middle), and density of labeled somata in the MCL (right). ***E***, Comparison of labeling patterns between *Tbx21-Cre::Ai14*, *Ra13-Cre::Ai14*, and *Lbhd2-CreERT2::Ai14* lines. Left, Labeled cells in the MCL as a percentage of total number of labeled cells. Middle, tdTomato signal density in the upper half of EPL subtracted by the signal density in the lower half of EPL. Right, Number of labeled cells detected per mm of MCL. *N* = 3 mice per transgenic line for all plots. Data are mean ± SEM. Statistical significance: *p = 0.05; **p = 0.01. For details, see Experimental design and statistical analysis.

The developmental accumulation described above makes *Ra13-Cre* unsuitable for investigating MCs in adult mice. However, the ISH signal for *Lbhd2* mRNA indicates a clear preference for MCs in adulthood. Therefore, it is possible that, when the recombination efficiency is calibrated appropriately, a more selective labeling of MCs may be feasible. To this end, we generated a new knock-in line (Fig. 7*A*) using CRISPR/Cas9, where the inducible Cre-recombinase, Cre*-ERT2* (Feil et al., 1997), is inserted into the 3′-UTR of the *Lbhd2* gene (the target sequence: 5′-ACCAAGAGGACCTCCAT-3′; Fig. 7*A*).

**Figure 8.**
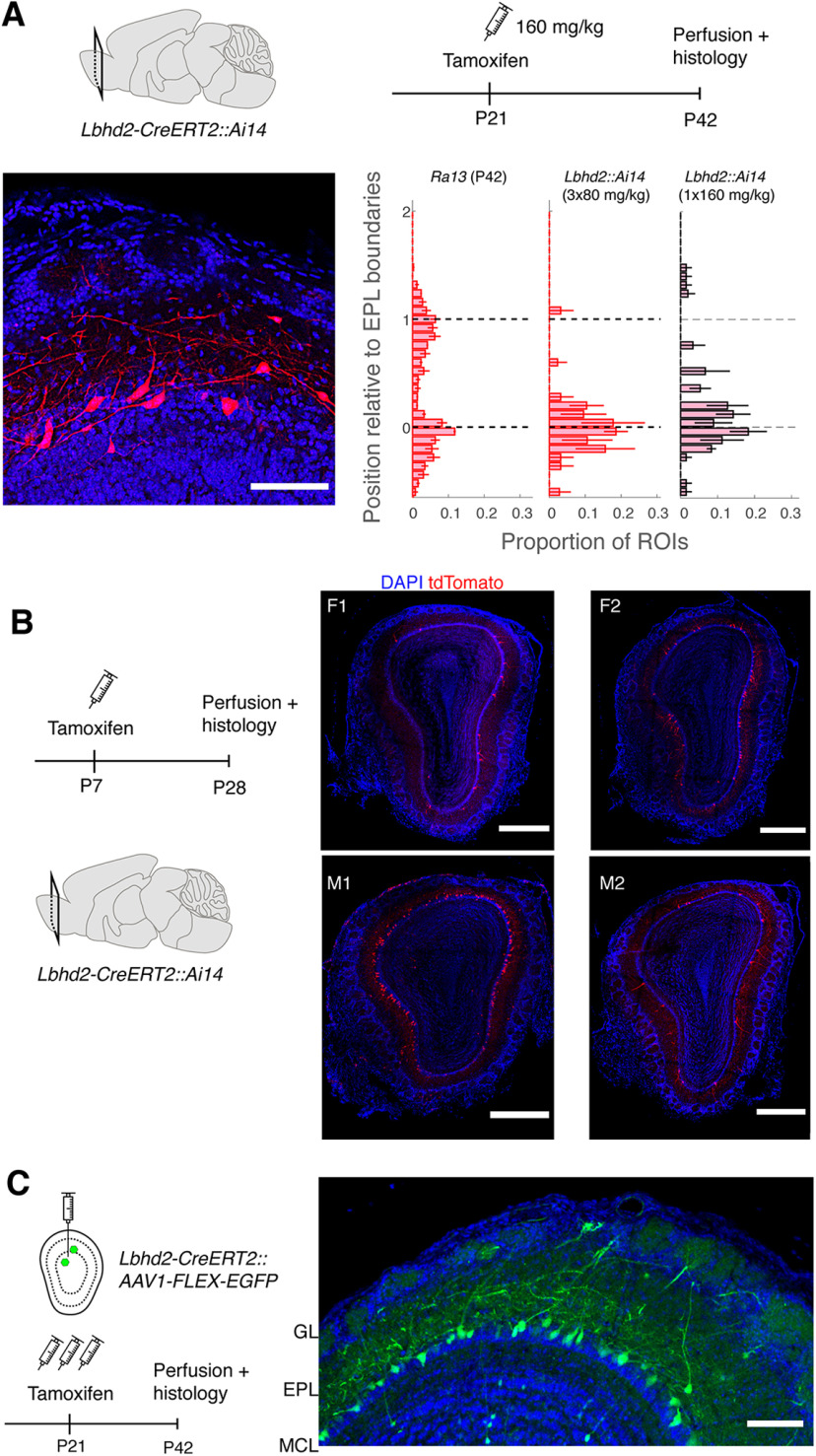
Further characterization of tamoxifen-dependent recombination. ***A***, Recombination pattern after 1× 160 mg/kg at P21, with tdTomato analyzed at P42. Scale bar, 0.1 mm. Bottom right, Summary of labeled soma positions relative to the EPL layers compared with *Ra13-Cre::Ai14* and *Lbhd2-CreERT2::Ai14* (3× 80 mg/kg). *N* = 3 mice. ***B***, Recombination pattern following tamoxifen injection at P7. *Lbhd2-CreERT2::Ai14* pups at postnatal day 7 were injected with a lowest dose tamoxifen (80 mg/kg intraperitoneally, once). Right, Recombination patterns from 2 females (F) and 2 males (M), as indicated. Scale bar, 0.5 mm. ***C***, Tamoxifen-induced recombination with AAV-mediated expression. Tamoxifen (3× 80 mg/kg) was administered intraperitoneally starting on the day of AAV (AAV1-flex-EGFP) injection in the dorsal OB and EGFP expression analyzed 3 weeks later. AAVs for conditional expression can exhibit Cre-independent, “leak expression” depending on the production protocol (Fischer et al., 2019) or if not diluted enough.

To test whether selective labeling is maintained beyond P7 in the new, inducible Cre-driver line, we injected tamoxifen intraperitoneally at P21 in *Lbhd2-CreERT2::Ai14* mice, and analyzed the distribution of red fluorescence 3 weeks after injection to assess the recombination pattern (Figs. 7*B–E*, 8*A*). At the lowest dose tested (one injection of 80 mg/kg), the labeling was sparse (average density of labeled MCs = 5.7 ± 0.9 cells per mm), but 83.7 ± 3.7% of labeled somata were located in the MCL (Fig. 7*C*,*D*). Other labeled cells were mostly TCs, save for sporadic labeling in granule cells, which constituted ∼1% of the labeled cells. When the dose was increased to three intraperitoneal injections of tamoxifen (at 80 mg/kg per day, over 3 d), denser labeling was achieved (mean density = 10 ± 4 cells per mm; 78.0 ± 11.0% of labeled somata were in MCL) while maintaining specificity, indicating that the tamoxifen dose can be calibrated to titrate the specificity and density of labeling. Compared with the patterns of recombination observed with existing lines, namely, *Tbx21-Cre* and *Ra13-Cre* mice, overall, the new line achieves a labeling that is substantially more selective for MCs, as measured by the positions of somata (*p* = 0.016, *F* = 6.01, one-way ANOVA; *n* = 3 mice for *Tbx21-Cre* and *Ra13-Cre*, 4 mice for *Lbhd2-IRES-CreERT2*) and dendrites (*p* = 2.99 × 10^−6^, *F* = 59.31, one-way ANOVA; *n* = 3 mice for *Tbx21-Cre* and *Ra13-Cre*, 4 mice for *Lbhd2-IRES-CreERT2*). Consistent with the recombination pattern observed with the *Ra13-Cre* line, tamoxifen injection at P7 also resulted in an MC-specific labeling (Fig. 8*B*). To test whether AAV-mediated conditional labeling is possible, AAV1-pCAG-Flex-EGFP-WPRE (100 nl) was stereotaxically injected into the dorsal OB at a depth of 300 µm below the brain surface in P21 *Lbhd2-CreERT2::Ai14* mice. Tamoxifen injections (3 × 80 mg/kg, i.p.) overlapped such that the first of the three injections occurred immediately after the AAV injection. Three weeks later, labeling pattern was analyzed, which showed predominantly MC-selective labeling similar to the pattern obtained with the *Ai14* reporter line (Fig. 8*C*).

**Figure 9.**
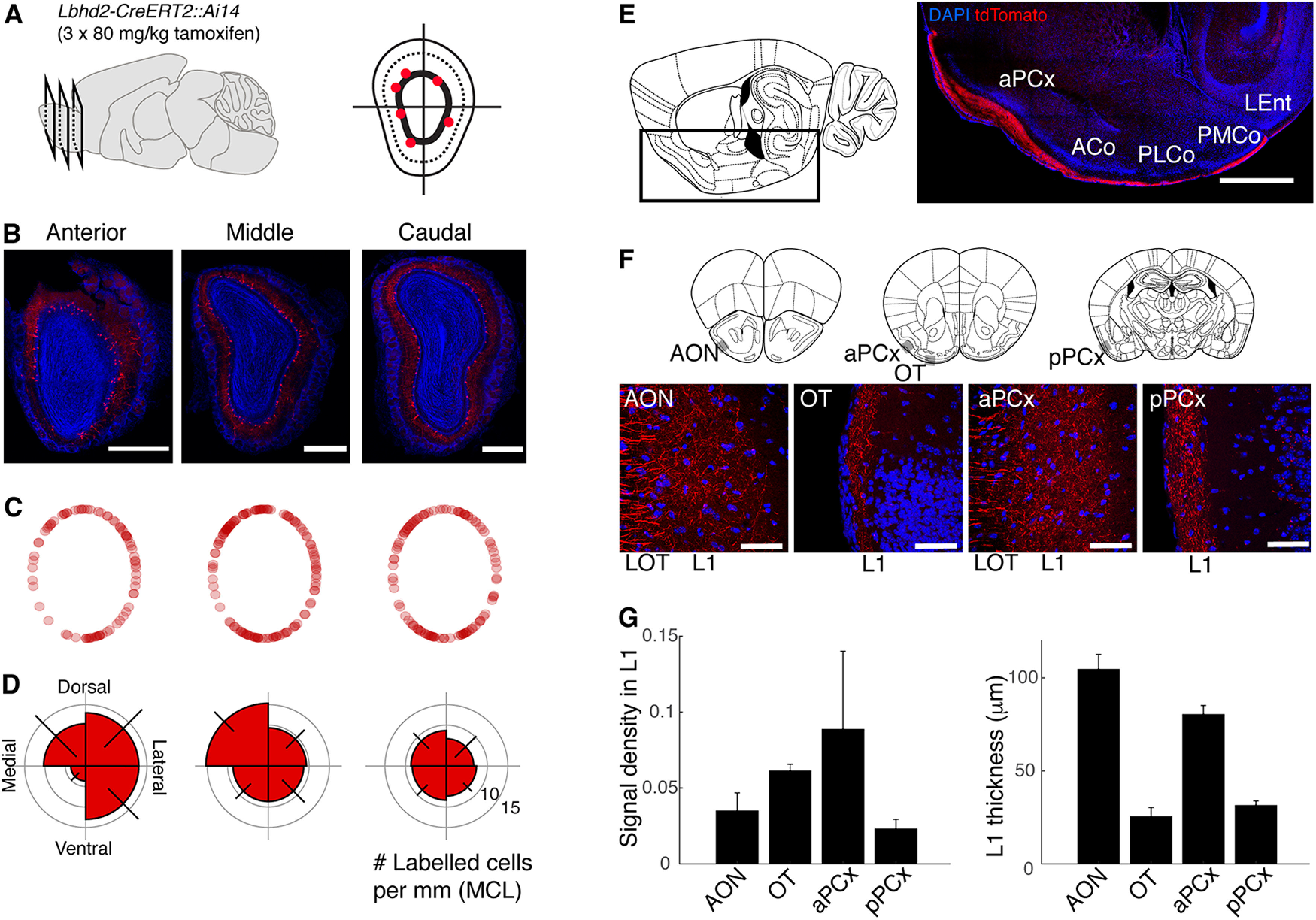
Properties of labeled MCs: OB domain-dependent variations and axon projection patterns. ***A–D***, Distribution of labeled MCs in the OB. ***A***, Schematic of analysis approach: positions of labeled MCs (red dots) were analyzed for anterior, middle, and caudal levels of the OB of *Lbhd2-CreERT2::Ai14* mice (tamoxifen dose = 3× 80 mg/kg). Angular positions were measured relative to the center of the OB. ***B***, Coronal OB images at each AP level from an example animal. Scale bar, 0.5 mm. ***C***, Positions of labeled MCs from all animals (one dot represents one labeled MC), projected on a standardized MCL position (see Materials and Methods). *N* = 3 mice. ***D***, Polar histograms showing the number of labeled MCs per mm of MCL for each quadrant. Error bar indicates SEM. ***E***, ***F***, Projection patterns of labeled fibers. ***E***, Image of a sagittal brain section (right) at the ML plane indicated in the illustration (left), with the imaged location marked by the black outline, showing tdTomato signal present in the lateral olfactory tract (LOT) and the molecular layer for the entire antero-caudal extent of the anterior piriform cortex (aPCx), anterior cortical amygdaloid nucleus (ACo), posterolateral cortical amygdaloid nucleus (PLCo), and posteromedial cortical amygdaloid nucleus (PMCo). Scale bar, 1 mm. ***F***, High magnification (40× objective) of coronal sections taken at the planes shown in illustrations (top). Gray boxes represent approximate location of images below. Labeled fibers appear as fascicles in the LOT, while thinner, densely present labeled fibers are visible in the superficial, molecular layer (L1) for the anterior olfactory nucleus (AON), olfactory tubercle (OT), and the anterior and posterior piriform cortices (aPCx and pPCx, respectively). Scale bar, 50 µm. ***G***, Summary quantification of signal density in the molecular layer for the 4 regions (left), and the thickness of the labeled L1 for the corresponding regions (right). *N* = 3 mice, one image plane each.

Having achieved MC-selective labeling with the *Lbhd2-CreERT2* line, we wished to further characterize the properties of the labeled cells in the OB, as well as the distribution of labeled fibers in the olfactory cortices (Fig. 9). Specifically, we wished to assess whether the labeled MCs are present uniformly in all domains of the OB. To this end, confocal images from coronal sections from anterior, middle, and caudal levels of the OB from *Lbhd2-CreERT2::Ai14* mice were analyzed. This revealed consistent MCL labeling in all regions of the OB, except for the most anterior level, which tended to show a sparser labeling on the medial side (Fig. 9*B–D*), although this was not statistically significant (*p* = 0.56, two-way ANOVA; *n* = 3 mice). In terms of the projection patterns of labeled fibers in the olfactory cortices, we detected red fluorescent fibers as fascicles throughout the antero-caudal extent of the lateral olfactory tract (Fig. 9*A*,*B*), as well as thin fibers with bouton-like structures in the molecular layers of olfactory cortices, including in the anterior olfactory nucleus, olfactory tubercle, and the anterior and posterior piriform cortices (Fig. 9*E–G*).

**Figure 10.**
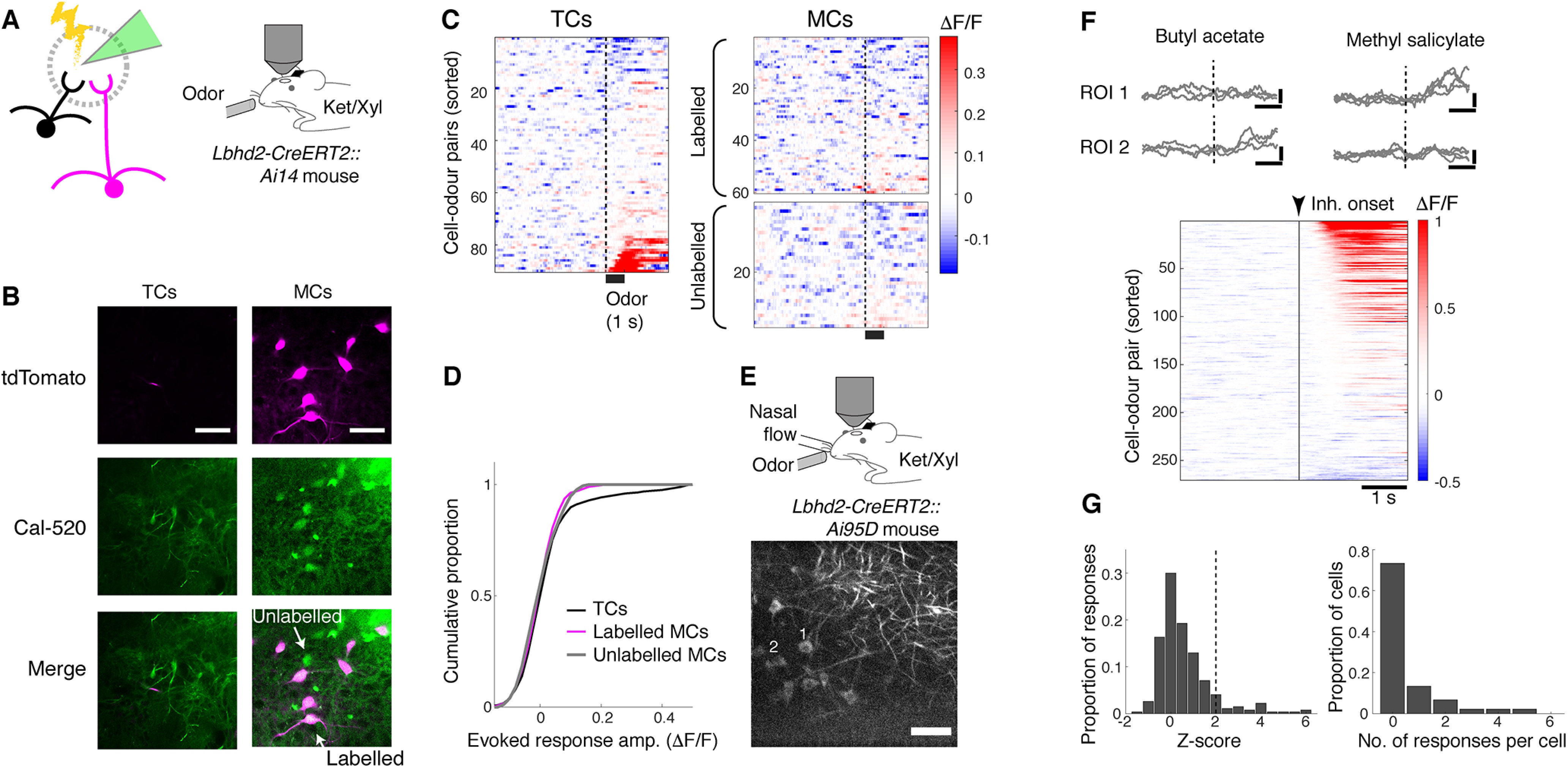
Odor response properties of labeled MCs compared with TCs. ***A***, Schematic showing low-intensity electroporation of Cal-520 dextran solution in the GL. ***B***, MCs were those located ∼300 µm below the brain surface, red fluorescence is pseudo-colored magenta (labeled represents red fluorescent cells + green fluorescence; unlabeled represents loaded cells without red fluorescence), whereas TCs were smaller cells located more superficially. Strongly fluorescent cells were excluded from analysis. Scale bar, 50 µm. ***C***, Normalized fluorescence (ΔF/F) from TCs (left) and labeled and unlabeled MCs (right, top and bottom, respectively), shown as color map (*n* = 2 mice). Excitatory responses are more prevalent in TCs. Cal-520 has a lower affinity to Ca^2+^ than GCaMP6 variants, which may make hyperpolarizing responses to odors less detectable. ***D***, Cumulative histogram of response amplitude, for TCs (black), labeled MCs (magenta), and unlabeled MCs (gray). Overall distributions are not significantly different (two-sample Kolmogorov–Smirnov test; *p* = 0.56). ***E***, Top, Experimental configuration. Bottom, Example FOV at a depth ∼280 µm below the brain surface. Scale bar, 50 µm. ***F***, Top, Example transients from two ROIs indicated in ***E***. Calibration: vertical, 0.5 ΔF/F; horizontal, 1 s. Dotted line indicates onset of the first inhalation after final valve opening. Bottom, Summary of fluorescence change in response to odor presentations shown with a color map (*n* = 228 cell-odor pairs, 38 cells, 3 mice). ***G***, Summary statistics of evoked responses. Left, Histogram of mean fluorescence change during odor (1 s from inhalation onset) expressed as *Z* score for the data in ***E***. Right, Number of odors that each cell responds with a fluorescence increase (*Z* score >2).

In addition to the anatomic traits, to assess the labeled cells functionally, we loaded a synthetic calcium indicator, Cal-520 dextran, by electroporation (Fig. 10*A*), using a low-intensity protocol (Hovis et al., 2010). Using two-photon microscopy in mice anesthetized with ketamine and xylazine, odor response properties of labeled MCs versus superficially located TCs were compared (Fig. 10*A*,*B*). As above, we used 42-d-old *Lbhd2-CreERT2::Ai14* mice, where recombination was induced with tamoxifen (3× 80 mg/kg) at P21. Consistent with previous reports (Nagayama et al., 2010; Burton and Urban, 2014; Ackels et al., 2020; Eiting and Wachowiak, 2020), odors excited TCs more than labeled or unlabeled MCs (Fig. 10*C*,*D*). No obvious difference was observed between labeled and unlabeled MCs. However, the lack of convincing responses raises some questions about the identity of labeled cells. Localized loading of dyes by electroporation allows a direct comparison of TCs and MCs belonging to the same glomerulus, but the low yield and the low sensitivity of the indicator are disadvantageous, especially when responses are sparse. To address this, we expressed GCaMP6f conditionally by crossing the *Lbhd2-CreERT2* line with the *Ai95D* line (Madisen et al., 2015), with tamoxifen injection at P21. Two weeks later, the injected mice were implanted with a cranial window over the left OB; and after 2 further weeks, the OB was imaged with a two-photon microscope under ketamine/xylazine anesthesia. On average, 5 or 6 fluorescent cells were visible in a given FOV (256 × 256 µm) at a depth ∼ 300 µm below the brain surface (Fig. 10*E*). This time, a fraction of cell-odor pairs (22.2%; *n* = 270 cell-odor pairs, 45 cells; 4 mice) exhibited robust fluorescence increases locked to odor presentations (Fig. 10*F*), although the majority of cells did not show detectable responses to any of the 6 odors presented (Fig. 10*G*). Overall, the results here indicate that the new inducible Cre-driver line, *Lbhd2-CreERT2*, achieves a highly specific labeling of functional MCs in the OB.

**Figure 11.**
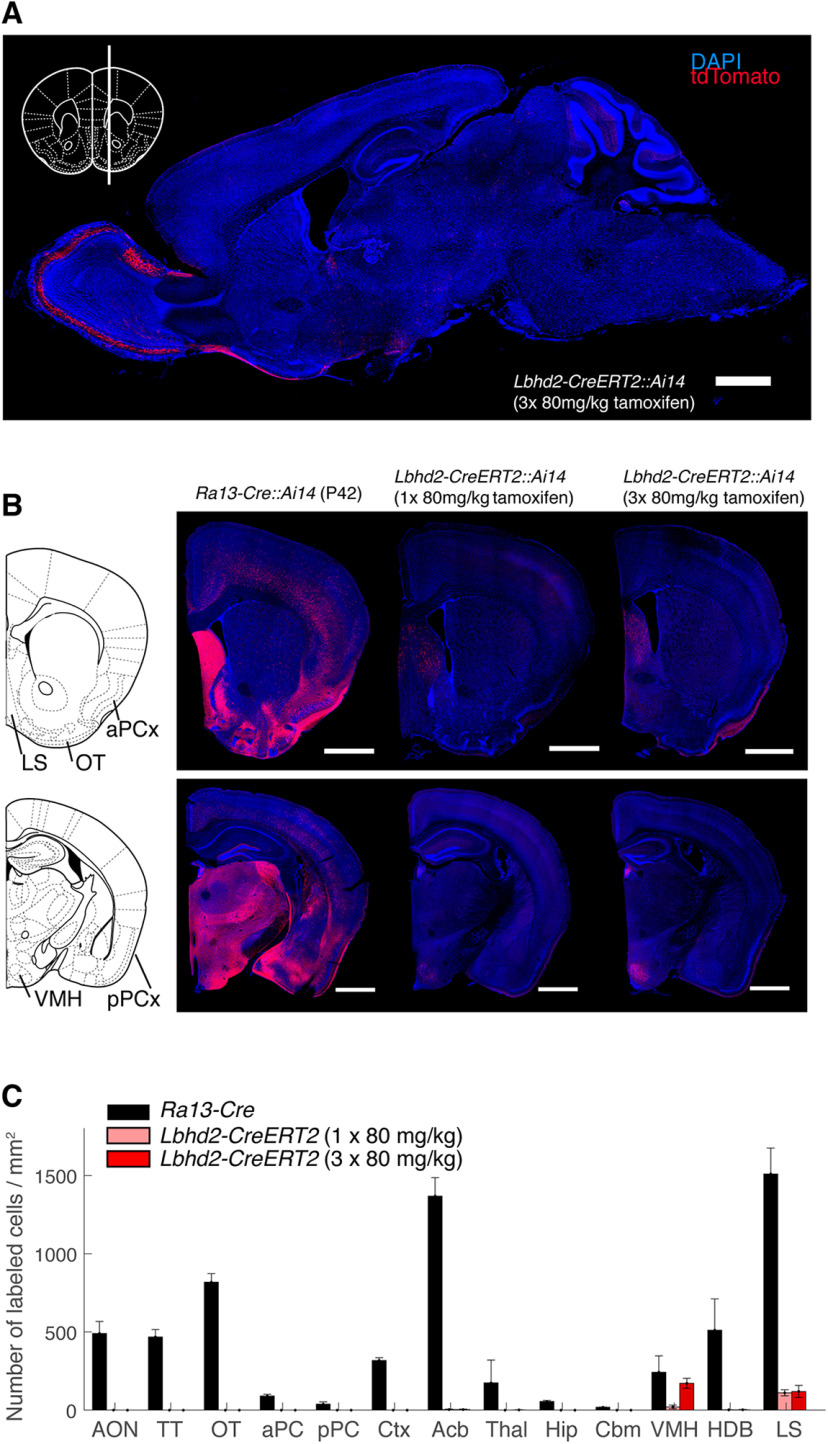
Brain-wide labeling is significantly reduced in the *Lbhd2-CreERT2* line. ***A***, A sagittal view of an example brain from a P42 *Lbhd2-CreERT2* mouse, which received 3 doses of tamoxifen (80 mg·kg^−1^) at P21, showing DAPI (blue) and tdTomato (red) signals. Inset, The ML plane for the sagittal section. Scale bar, 1 mm. ***B***, Example coronal images from *Ra13::Ai14* (left), and *Lbhd2-IRES-CreERT2::Ai14* mice that received 1× tamoxifen and 3× tamoxifen doses (middle and right, respectively). Left, Corresponding anatomic borders at this plane. Scale bar, 1 mm. ***C***, Summary showing average density of labeled cells for each anatomic region (*n* = 3 mice per region). Acb, Accumbens nucleus (shell); AON, anterior olfactory nucleus; Cbm, cerebellum; Ctx, cerebral cortex; aPCx, anterior piriform cortex; HDB, nucleus of the horizontal limb of the diagonal band; Hip, hippocampus; LS, lateral septum; OT, olfactory tubercle; pPCx, posterior piriform cortex; Thal, thalamus; TT, tenia tecta; VMH, ventromedial nucleus of the hypothalamus.

**Figure 12.**
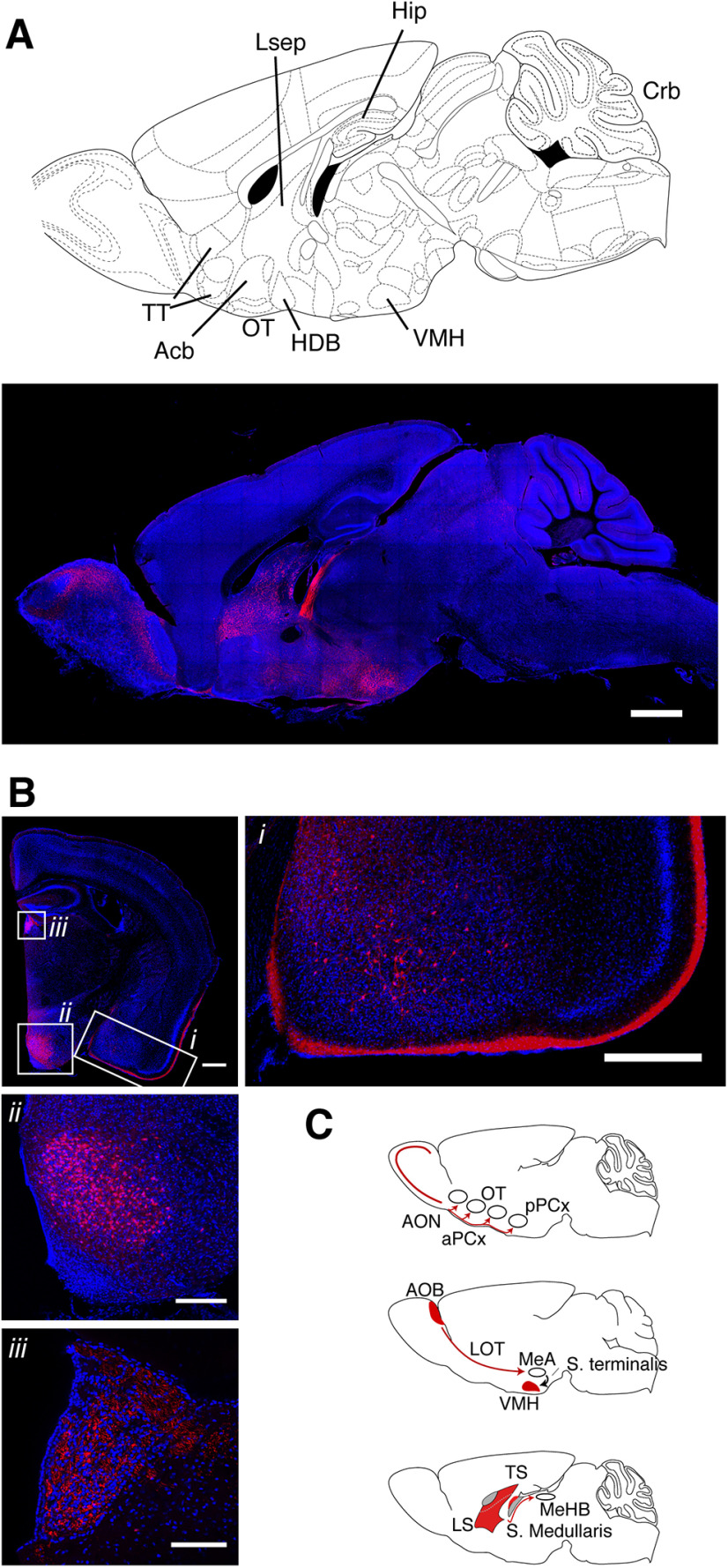
Recombination pattern outside of the OB in *Lbhd2-CreERT2* mice. ***A***, Confocal image at a sagittal plane ∼0.36 mm from the midline in a *Lbhd2-CreERT2::Ai14* mouse. Scale bar, 1 mm. ***B***, Coronal view at ∼1.46 mm posterior to the bregma, showing (***Bi***) labeled cells in the basolateral amygdaloid nucleus, fiber endings in the molecular layer medial amygdaloid nucleus and posterior piriform cortex; (***Bii***) densely labeled somata in the ventromedial nucleus of the hypothalamus; and (***Biii***) labeled fibers in the medial habenular nucleus. ***C***, Summary of labeled structures with respect to distinct pathways. Top, MCs of the main OB are labeled, but not their cortical targets. Middle, Principal neurons of the accessory OB are labeled. Labeled fibers, but not somata, are visible in the medial amygdaloid nucleus. The target of the medial amygdaloid nucleus, namely, the ventromedial nucleus of the hypothalamus, has densely labeled cells. Bottom, Lateral septum densely contains labeled cells; the output fiber tracts are strongly labeled (stria medullaris), and labeled fibers are clearly visible in the target structure, namely, the medial habenular nucleus.

Finally, we examined the recombination pattern in the brain at large, beyond the OB. To assess this, we analyzed the distribution of labeled somata in the anterior olfactory nucleus, olfactory tubercle, anterior and posterior piriform cortex, and tenia tacta, as well as other, commonly studied regions, including the thalamus, cerebellum, hippocampus, and cerebral cortex. In the *Lbhd2-CreERT2* mice, for both doses of tamoxifen tested, consistent labeling was observed unexpectedly in a small number of nuclei, including the ventromedial nucleus of the hypothalamus and the lateral septum (Figs. 11, 12). Compared with the *Ra13-Cre* driver line, the olfactory cortices were devoid of fluorescent cells (Fig. 11*B,C*; no labeled cells were detected for aPCx, pPCx, AON, and OT in *Lbhd2- CreERT2::Ai14* mice; in *Ra13*::*Ai14* mice, mean density of labeled cells = 91.2, 38.5, 490.2, and 818.0 labeled cells per mm^2^ for aPCx, pPCx, AON, and OT, respectively; *p* = 1.03 × 10^−38^, *F* = 295.04, two-way ANOVA, with mean densities for *Lbhd2-CreERT2* groups were significantly different from *Ra13-Cre*; *n* = 3 mice per group). Thus, the results indicate that the labeling is, overall, relatively specific to MCs in the whole brain, suggesting that the new inducible Cre-driver line may be suitable for a variety of studies to investigate olfactory processing.

## Discussion

A wide variety of neuron types that exist in the sensory systems are thought to reflect diverse components for information processing in the brain (Masland, 2004; Luo et al., 2018). Availability of Cre-driver lines have led to a multitude of fundamental insights into unique, cell type-specific contributions to sensory processing and perception (Münch et al., 2009; Dhande et al., 2013; Cruz-Martín et al., 2014; Takahashi et al., 2020). Recent progress in the acquisition, analyses, and applications of large-scale gene expression data have allowed efficient analysis of the differences between cell types of interest (Birnbaum, 2018; Luo et al., 2018). In this study, we used a publicly available gene expression dataset to discover candidate molecular markers for the key second-order cells of the olfactory system, MCs, which we validated with histology, and finally with new Cre-driver lines generated by CRISPR/Cas9-mediated gene editing. We report that one driver line in particular provides a substantial improvement in the ability to selectively label MCs.

Among the several candidates identified from our differential expression analysis, we found *Lbhd2* to be the most promising. Specifically, at postnatal day 7, the recombination pattern for the noninducible, *Ra13-Cre::Ai14* is restricted mainly to MCs in the OB. This pattern is consistent with the description on the GENSAT expression database (Gong et al., 2007). Since TCs are already present and located superficially in the EPL at this stage of development (Mizuguchi et al., 2012), this pattern likely reflects genuine MC specificity in neonatal mice. Despite an increase in the sporadic *Lbhd2*-driven labeling of TCs and other regions of the brain at later stages, the preferential expression in MCs over TCs that persists in adulthood can be used to our advantage. Thus, with our new inducible Cre-driver line, the expression can be targeted selectively to MCs even in adulthood. It is notable that, despite the fact that many genes were differentially expressed across the two cell types, markers suitable as genetic tools are harder to identify, especially when selective expression is required across developmental stages. This difficulty may partly be because of the similarity between MCs and TCs, and that cell types are often defined by a combination of genes, rather than single genes (Luo et al., 2018).

In the OB, labeled MCs were present in most domains of the OB, except for a small patch on the medial, anterior OB that showed a curious lack of labeling. Whether or not these correspond to subclasses of MCs, for example, those that differ in the glomerular association (Li et al., 2017), or cortical projection patterns (Zeppilli et al., 2020), will be intriguing for future investigation. Outside of the OB, labeled somata were sparse, if not absent, especially in the areas that MCs target, including in the anterior olfactory nucleus, olfactory tubercle, and the anterior and posterior piriform cortices. This makes the *Lbhd2-CreERT2* line suitable for investigating the downstream, decoding mechanisms of mitral-specific activity in all these areas, and also when imaging from boutons of MCs (Pashkovski et al., 2020). Beyond these areas, however, we observed a small number of specific regions that showed the presence of labeled somata. The areas include the lateral septum, ventromedial nucleus of the hypothalamus, and the medial amygdala, even at the lowest dose of tamoxifen used. Thus, future studies using this line need to take this into account when interpreting data, in particular for investigating innate, social behavior, which involve these areas (Stowers and Liberles, 2016). However, the fact that only a subset of the nuclei in the pathways are labeled may make this line unexpectedly useful for investigating mechanisms of social behavior.

This study was aided by publicly accessible data, speeding up discovery. One limitation, if any, in using this dataset for this study may have been the data size, where only a small fraction of OB cells expressed *Tbx21*, and even fewer belonged to the putative MC cluster. The relatively small MC cluster size may partly be biological. Of the neurons present in the OB, ∼80% are GABAergic. Further, glutamatergic neurons comprise heterogeneous groups, including those that lack lateral dendrites (Hayar et al., 2004; Antal et al., 2006). Thus, MCs comprise only a small proportion (∼1%) of OB neurons (Burton, 2017; Schwarz et al., 2018). Our histology indicates that superficially located, *Tbx21*-expressing cells are located below the GL, unlike the *Cck*-expressing population that includes a dense population located more superficially. It should also be noted that a large proportion of glutamatergic and *Cck*-expressing cells were found outside of the *Tbx21*-positive cluster. Some of this latter group may correspond to external TCs, which are glutamatergic but lack lateral dendrites (Macrides and Schneider, 1982). It is possible that protocols used to obtain the scRNA-seq data may have been inadvertently biased against large cells with prominent dendrites, such as the filtering step involving a pore size of 30 µm (Zeisel et al., 2018).

Despite the need for tamoxifen, this new method for labeling MCs has several advantages over the existing methods. Currently, MC labeling and manipulations are achieved predominantly by depth, birthdate, or retrograde viral expression using the differential projection targets of MCs versus TCs (Haberly and Price, 1977; Imamura et al., 2011; Rothermel et al., 2013; Economo et al., 2016). While this can indeed bias expression patterns, the overlap in somatic and dendritic locations (Schwarz et al., 2018), as well as projection targets (Haberly and Price, 1977; Igarashi et al., 2012), means that it is not trivial to achieve a highly selective labeling. In contrast, our transgenic mouse line described here allows for reproducible and selective labeling of MCs over TCs, with the added advantage that labeled MCs are located throughout the OB. Even in imaging applications that can distinguish the cell type based on the soma depth, with the new driver line, it will be possible to investigate the physiology of subcellular compartments, such as the long lateral dendrites, without the need to painstakingly trace back the structures to somata for cell type identification. Similarly, investigations of downstream decoding mechanisms, such as one involving precise optogenetic activations of OB projections using patterned light stimuli (Chong et al., 2020), may now be done in a cell type-specific manner. Thus, our new tool may bring us closer to understanding how parallel olfactory processing contributes to mechanisms of sensory perception and, ultimately, behavior.

## References

[B1] Ackels T, Jordan R, Schaefer AT, Fukunaga I (2020) Respiration-locking of olfactory receptor and projection neurons in the mouse olfactory bulb and its modulation by brain state. Front Cell Neurosci 14:220. 10.3389/fncel.2020.00220 32765224 PMC7378796

[B2] Antal M, Eyre M, Finklea B, Nusser Z (2006) External tufted cells in the main olfactory bulb form two distinct subpopulations. Eur J Neurosci 24:1124–1136. 10.1111/j.1460-9568.2006.04988.x 16930438 PMC1557706

[B3] Birnbaum KD (2018) Power in numbers: single-cell RNA-Seq strategies to dissect complex tissues. Annu Rev Genet 52:203–221. 10.1146/annurev-genet-120417-031247 30192636 PMC6314027

[B4] Bochkov YA, Palmenberg AC (2006) Translational efficiency of EMCV IRES in bicistronic vectors is dependent upon IRES sequence and gene location. Biotechniques 41:283–292. 10.2144/000112243 16989088

[B5] Burton SD (2017) Inhibitory circuits of the mammalian main olfactory bulb. J Neurophysiol 118:2034–2051. 10.1152/jn.00109.2017 28724776 PMC5626887

[B6] Burton SD, Urban NN (2014) Greater excitability and firing irregularity of tufted cells underlies distinct afferent-evoked activity of olfactory bulb mitral and tufted cells. J Physiol 592:2097–2118. 10.1113/jphysiol.2013.269886 24614745 PMC4227897

[B7] Campello RJ, Moulavi D, Sander J (2013) Density-based clustering based on hierarchical density estimates. In: Advances in knowledge discovery and data mining (Pei J, Tseng VS, Cao L, Motoda H, Xu G, eds), pp 160–172. Berlin: Springer.

[B8] Chong E, Moroni M, Wilson C, Shoham S, Panzeri S, Rinberg D (2020) Manipulating synthetic optogenetic odors reveals the coding logic of olfactory perception. Science 368:eaba2357. 10.1126/science.aba2357 32554567 PMC8237706

[B9] Cong L, Ran FA, Cox D, Lin S, Barretto R, Habib N, Hsu PD, Wu X, Jiang W, Marraffini LA, Zhang F (2013) Multiplex genome engineering using CRISPR/Cas systems. Science 339:819. 10.1126/science.1231143 23287718 PMC3795411

[B10] Cruz-Martín A, El-Danaf RN, Osakada F, Sriram B, Dhande OS, Nguyen PL, Callaway EM, Ghosh A, Huberman AD (2014) A dedicated circuit links direction-selective retinal ganglion cells to the primary visual cortex. Nature 507:358–361. 10.1038/nature12989 24572358 PMC4143386

[B11] Daigle TL, Madisen L, Hage TA, Valley MT, Knoblich U, Larsen RS, Takeno MM, Huang L, Gu H, Larsen R, Mills M, Bosma-Moody A, Siverts LA, Walker M, Graybuck LT, Yao Z, Fong O, Nguyen TN, Garren E, Lenz GH, et al. (2018) A suite of transgenic driver and reporter mouse lines with enhanced brain-cell type targeting and functionality. Cell 174:465–480.e422. 10.1016/j.cell.2018.06.035 30007418 PMC6086366

[B12] Dhande OS, Estevez ME, Quattrochi LE, El-Danaf RN, Nguyen PL, Berson DM, Huberman AD (2013) Genetic dissection of retinal inputs to brainstem nuclei controlling image stabilization. J Neurosci 33:17797–17813. 10.1523/JNEUROSCI.2778-13.2013 24198370 PMC3818553

[B13] Economo MN, Hansen KR, Wachowiak M (2016) Control of mitral/tufted cell output by selective inhibition among olfactory bulb glomeruli. Neuron 91:397–411. 10.1016/j.neuron.2016.06.001 27346531 PMC6474342

[B14] Eiting TP, Wachowiak M (2020) Differential impacts of repeated sampling on odor representations by genetically-defined mitral and tufted cell subpopulations in the mouse olfactory bulb. J Neurosci 40:6177–6188. 10.1523/JNEUROSCI.0258-20.2020 32601245 PMC7406285

[B15] Faedo A, Ficara F, Ghiani M, Aiuti A, Rubenstein JLR, Bulfone A (2002) Developmental expression of the T-box transcription factor T-bet/Tbx21 during mouse embryogenesis. Mech Dev 116:157–160. 10.1016/S0925-4773(02)00114-4 12128215

[B16] Feil R, Wagner J, Metzger D, Chambon P (1997) Regulation of Cre recombinase activity by mutated estrogen receptor ligand-binding domains. Biochem Biophys Res Commun 237:752–757. 10.1006/bbrc.1997.7124 9299439

[B17] Fischer KB, Collins HK, Callaway EM (2019) Sources of off-target expression from recombinase-dependent AAV vectors and mitigation with cross-over insensitive ATG-out vectors. Proc Natl Acad Sci USA 116:27001–27010. 10.1073/pnas.1915974116 PMC6936690 31843925

[B18] Fukunaga I, Berning M, Kollo M, Schmaltz A, Schaefer AT (2012) Two distinct channels of olfactory bulb output. Neuron 75:320–329. 10.1016/j.neuron.2012.05.017 22841316

[B19] Geramita MA, Burton SD, Urban NN (2016) Distinct lateral inhibitory circuits drive parallel processing of sensory information in the mammalian olfactory bulb. Elife 5:e16039. 10.7554/Elife.16039 27351103 PMC4972542

[B20] Gong S, Zheng C, Doughty ML, Losos K, Didkovsky N, Schambra UB, Nowak NJ, Joyner A, Leblanc G, Hatten ME, Heintz N (2003) A gene expression atlas of the central nervous system based on bacterial artificial chromosomes. Nature 425:917–925. 10.1038/nature02033 14586460

[B21] Gong S, Doughty M, Harbaugh CR, Cummins A, Hatten ME, Heintz N, Gerfen CR (2007) Targeting Cre recombinase to specific neuron populations with bacterial artificial chromosome constructs. J Neurosci 27:9817–9823. 10.1523/JNEUROSCI.2707-07.2007 17855595 PMC6672645

[B22] Haberly LB, Price JL (1977) The axonal projection patterns of the mitral and tufted cells of the olfactory bulb in the rat. Brain Res 129:152–157. 10.1016/0006-8993(77)90978-7 68803

[B23] Haddad R, Lanjuin A, Madisen L, Zeng H, Murthy VN, Uchida N (2013) Olfactory cortical neurons read out a relative time code in the olfactory bulb. Nat Neurosci 16:949–957. 10.1038/nn.3407 23685720 PMC3695490

[B24] Hayar A, Karnup S, Ennis M, Shipley MT (2004) External tufted cells: a major excitatory element that coordinates glomerular activity. J Neurosci 24:6676–6685. 10.1523/JNEUROSCI.1367-04.2004 15282270 PMC6729710

[B25] Heintz N (2004) Gene Expression Nervous System Atlas (GENSAT). Nat Neurosci 7:483. 10.1038/nn0504-483 15114362

[B26] Hovis KR, Padmanabhan K, Urban NN (2010) A simple method of in vitro electroporation allows visualization, recording, and calcium imaging of local neuronal circuits. J Neurosci Methods 191:1–10. 10.1016/j.jneumeth.2010.05.017 20669363 PMC2974945

[B27] Igarashi KM, Ieki N, An M, Yamaguchi Y, Nagayama S, Kobayakawa K, Kobayakawa R, Tanifuji M, Sakano H, Chen WR, Mori K (2012) Parallel mitral and tufted cell pathways route distinct odor information to different targets in the olfactory cortex. J Neurosci 32:7970–7985. 10.1523/JNEUROSCI.0154-12.2012 22674272 PMC3636718

[B28] Imamura F, Ayoub AE, Rakic P, Greer CA (2011) Timing of neurogenesis is a determinant of olfactory circuitry. Nat Neurosci 14:331–337. 10.1038/nn.2754 21297629 PMC3046046

[B29] Jordan R, Fukunaga I, Kollo M, Schaefer AT (2018) Active sampling state dynamically enhances olfactory bulb odor representation. Neuron 98:1214–1228.e1215. 10.1016/j.neuron.2018.05.016 29861286 PMC6030445

[B30] Kapoor V, Provost AC, Agarwal P, Murthy VN (2016) Activation of raphe nuclei triggers rapid and distinct effects on parallel olfactory bulb output channels. Nat Neurosci 19:271–282. 10.1038/nn.4219 26752161 PMC4948943

[B31] Kobak D, Berens P (2019) The art of using t-SNE for single-cell transcriptomics. Nat Commun 10:5416. 10.1038/s41467-019-13056-x 31780648 PMC6882829

[B32] Koldaeva A, Schaefer AT, Fukunaga I (2019) Rapid task-dependent tuning of the mouse olfactory bulb. eLife 8:e43558. 30724732 10.7554/eLife.43558 PMC6365054

[B33] Larsson LI, Rehfeld JF (1979) Localization and molecular heterogeneity of cholecystokinin in the central and peripheral nervous system. Brain Res 165:201–218. 10.1016/0006-8993(79)90554-7 369662

[B34] Lein ES, Hawrylycz MJ, Ao N, Ayres M, Bensinger A, Bernard A, Boe AF, Boguski MS, Brockway KS, Byrnes EJ, Chen L, Chen L, Chen TM, Chin MC, Chong J, Crook BE, Czaplinska A, Dang CN, Datta S, Dee NR, et al. (2007) Genome-wide atlas of gene expression in the adult mouse brain. Nature 445:168–176. 10.1038/nature05453 17151600

[B35] Lein ES, Borm LE, Linnarsson S (2017) The promise of spatial transcriptomics for neuroscience in the era of molecular cell typing. Science 358:64–69. 10.1126/science.aan6827 28983044

[B36] Li H, Horns F, Wu B, Xie Q, Li J, Li T, Luginbuhl DJ, Quake SR, Luo L (2017) Classifying *Drosophila* olfactory projection neuron subtypes by single-cell RNA sequencing. Cell 171:1206–1220.e1222. 10.1016/j.cell.2017.10.019 29149607 PMC6095479

[B37] Lim L, Pakan JM, Selten MM, Marques-Smith A, Llorca A, Bae SE, Rochefort NL, Marín O (2018) Optimization of interneuron function by direct coupling of cell migration and axonal targeting. Nat Neurosci 21:920–931. 10.1038/s41593-018-0162-9 29915195 PMC6061935

[B38] Luo L, Callaway EM, Svoboda K (2018) Genetic dissection of neural circuits: a decade of progress. Neuron 98:256–281. 10.1016/j.neuron.2018.03.040 29673479 PMC5912347

[B39] Macrides F, Schneider SP (1982) Laminar organization of mitral and tufted cells in the main olfactory bulb of the adult hamster. J Comp Neurol 208:419–430. 10.1002/cne.902080410 7119169

[B40] Madisen L, Zwingman TA, Sunkin SM, Oh SW, Zariwala HA, Gu H, Ng LL, Palmiter RD, Hawrylycz MJ, Jones AR, Lein ES, Zeng H (2010) A robust and high-throughput Cre reporting and characterization system for the whole mouse brain. Nat Neurosci 13:133–140. 10.1038/nn.2467 20023653 PMC2840225

[B41] Madisen L, Mao T, Koch H, Zhuo JM, Berenyi A, Fujisawa S, Hsu YW, Garcia AJ, Gu X, Zanella S, Kidney J, Gu H, Mao Y, Hooks BM, Boyden ES, Buzsáki G, Ramirez JM, Jones AR, Svoboda K, Han X, et al. (2012) A toolbox of Cre-dependent optogenetic transgenic mice for light-induced activation and silencing. Nat Neurosci 15:793–802. 10.1038/nn.3078 22446880 PMC3337962

[B42] Madisen L, Garner AR, Shimaoka D, Chuong AS, Klapoetke NC, Li L, van der Bourg A, Niino Y, Egolf L, Monetti C, Gu H, Mills M, Cheng A, Tasic B, Nguyen TN, Sunkin SM, Benucci A, Nagy A, Miyawaki A, Helmchen F, et al. (2015) Transgenic mice for intersectional targeting of neural sensors and effectors with high specificity and performance. Neuron 85:942–958. 10.1016/j.neuron.2015.02.022 25741722 PMC4365051

[B43] Masland RH (2004) Neuronal cell types. Curr Biol 14:R497–R500. 10.1016/j.cub.2004.06.035 15242626

[B44] Mitsui S, Igarashi KM, Mori K, Yoshihara Y (2011) Genetic visualization of the secondary olfactory pathway in Tbx21 transgenic mice. Neural Syst Circuits 1:5. 10.1186/2042-1001-1-5 22330144 PMC3257540

[B45] Mizuguchi R, Naritsuka H, Mori K, Mao CA, Klein WH, Yoshihara Y (2012) Tbr2 deficiency in mitral and tufted cells disrupts excitatory–inhibitory balance of neural circuitry in the mouse olfactory bulb. J Neurosci 32:8831–8844. 10.1523/JNEUROSCI.5746-11.2012 22745484 PMC3700647

[B46] Mizuno S, Dinh TT, Kato K, Mizuno-Iijima S, Tanimoto Y, Daitoku Y, Hoshino Y, Ikawa M, Takahashi S, Sugiyama F, Yagami K (2014) Simple generation of albino C57BL/6J mice with G291T mutation in the tyrosinase gene by the CRISPR/Cas9 system. Mamm Genome 25:327–334. 10.1007/s00335-014-9524-0 24879364

[B47] Mori K, Kishi K, Ojima H (1983) Distribution of dendrites of mitral, displaced mitral, tufted, and granule cells in the rabbit olfactory bulb. J Comp Neurol 219:339–355. 10.1002/cne.902190308 6619342

[B48] Münch TA, da Silveira RA, Siegert S, Viney TJ, Awatramani GB, Roska B (2009) Approach sensitivity in the retina processed by a multifunctional neural circuit. Nat Neurosci 12:1308–1316. 10.1038/nn.2389 19734895

[B49] Nagai Y, Sano H, Yokoi M (2005) Transgenic expression of Cre recombinase in mitral/tufted cells of the olfactory bulb. Genesis 43:12–16. 10.1002/gene.20146 16106355

[B50] Nagayama S, Enerva A, Fletcher M, Masurkar A, Igarashi K, Mori K, Chen W (2010) Differential axonal projection of mitral and tufted cells in the mouse main olfactory system. Front Neural Circuits 4:120. 10.3389/fncir.2010.00120 20941380 PMC2952457

[B51] Otazu GH, Chae H, Davis MB, Albeanu DF (2015) Cortical feedback decorrelates olfactory bulb output in awake mice. Neuron 86:1461–1477. 10.1016/j.neuron.2015.05.023 26051422 PMC7448302

[B52] Papaioannou VE, Silver LM (1998) The T-box gene family. BioEssays 20:9–19. 10.1002/(SICI)1521-1878(199801)20:1<9::AID-BIES4>3.0.CO;2-Q 9504043

[B53] Pashkovski SL, Iurilli G, Brann D, Chicharro D, Drummey K, Franks KM, Panzeri S, Datta SR (2020) Structure and flexibility in cortical representations of odour space. Nature 583:253–258. 10.1038/s41586-020-2451-1 32612230 PMC7450987

[B54] Pfeffer CK, Xue M, He M, Huang ZJ, Scanziani M (2013) Inhibition of inhibition in visual cortex: the logic of connections between molecularly distinct interneurons. Nat Neurosci 16:1068–1076. 10.1038/nn.3446 23817549 PMC3729586

[B55] Phillips ME, Sachdev RN, Willhite DC, Shepherd GM (2012) Respiration drives network activity and modulates synaptic and circuit processing of lateral inhibition in the olfactory bulb. J Neurosci 32:85–98. 10.1523/JNEUROSCI.4278-11.2012 22219272 PMC3566643

[B56] Rothermel M, Brunert D, Zabawa C, Díaz-Quesada M, Wachowiak M (2013) Transgene expression in target-defined neuron populations mediated by retrograde infection with adeno-associated viral vectors. J Neurosci 33:15195–15206. 10.1523/JNEUROSCI.1618-13.2013 24048849 PMC3776063

[B57] Sanes JR, Masland RH (2015) The types of retinal ganglion cells: current status and implications for neuronal classification. Annu Rev Neurosci 38:221–246. 10.1146/annurev-neuro-071714-034120 25897874

[B58] Schwarz D, Kollo M, Bosch C, Feinauer C, Whiteley I, Margrie TW, Cutforth T, Schaefer AT (2018) Architecture of a mammalian glomerular domain revealed by novel volume electroporation using nanoengineered microelectrodes. Nat Commun 9:183. 10.1038/s41467-017-02560-7 29330458 PMC5766516

[B59] Seroogy KB, Brecha N, Gall C (1985) Distribution of cholecystokinin-like immunoreactivity in the rat main olfactory bulb. J Comp Neurol 239:373–383. 10.1002/cne.902390403 2864364

[B60] Shekhar K, Lapan SW, Whitney IE, Tran NM, Macosko EZ, Kowalczyk M, Adiconis X, Levin JZ, Nemesh J, Goldman M, McCarroll SA, Cepko CL, Regev A, Sanes JR (2016) Comprehensive classification of retinal bipolar neurons by single-cell transcriptomics. Cell 166:1308–1323.e1330. 10.1016/j.cell.2016.07.054 27565351 PMC5003425

[B61] Short SM, Wachowiak M (2019) Temporal dynamics of inhalation-linked activity across defined subpopulations of mouse olfactory bulb neurons imaged in vivo. eneuro 6:ENEURO.0189-19.2019. 10.1523/ENEURO.0189-19.2019 PMC6597857 31209151

[B62] Ståhl PL, Salmén F, Vickovic S, Lundmark A, Navarro JF, Magnusson J, Giacomello S, Asp M, Westholm JO, Huss M, Mollbrink A, Linnarsson S, Codeluppi S, Borg Å, Pontén F, Costea PI, Sahlén P, Mulder J, Bergmann O, Lundeberg J, et al. (2016) Visualization and analysis of gene expression in tissue sections by spatial transcriptomics. Science 353:78–82. 10.1126/science.aaf2403 27365449

[B63] Stowers L, Liberles SD (2016) State-dependent responses to sex pheromones in mouse. Curr Opin Neurobiol 38:74–79. 10.1016/j.conb.2016.04.001 27093585 PMC4921285

[B64] Sugino K, Hempel CM, Miller MN, Hattox AM, Shapiro P, Wu C, Huang ZJ, Nelson SB (2006) Molecular taxonomy of major neuronal classes in the adult mouse forebrain. Nat Neurosci 9:99–107. 10.1038/nn1618 16369481

[B65] Takahashi N, Ebner C, Sigl-Glöckner J, Moberg S, Nierwetberg S, Larkum ME (2020) Active dendritic currents gate descending cortical outputs in perception. Nat Neurosci 23:1277–1285. 10.1038/s41593-020-0677-8 32747790

[B66] Tang F, Barbacioru C, Wang Y, Nordman E, Lee C, Xu N, Wang X, Bodeau J, Tuch BB, Siddiqui A, Lao K, Surani MA (2009) mRNA-Seq whole-transcriptome analysis of a single cell. Nat Methods 6:377–382. 10.1038/nmeth.1315 19349980

[B67] Taniguchi H, He M, Wu P, Kim S, Paik R, Sugino K, Kvitsiani D, Kvitsani D, Fu Y, Lu J, Lin Y, Miyoshi G, Shima Y, Fishell G, Nelson SB, Huang ZJ (2011) A resource of Cre driver lines for genetic targeting of GABAergic neurons in cerebral cortex. Neuron 71:995–1013. 10.1016/j.neuron.2011.07.026 21943598 PMC3779648

[B68] Tasic B, Menon V, Nguyen TN, Kim TK, Jarsky T, Yao Z, Levi B, Gray LT, Sorensen SA, Dolbeare T, Bertagnolli D, Goldy J, Shapovalova N, Parry S, Lee C, Smith K, Bernard A, Madisen L, Sunkin SM, Hawrylycz M, et al. (2016) Adult mouse cortical cell taxonomy revealed by single cell transcriptomics. Nat Neurosci 19:335–346. 10.1038/nn.4216 26727548 PMC4985242

[B69] van der Maaten L, Hinton G (2008) Visualizing data using t-SNE. J Mach Learn Res 9:2579–2605.

[B70] Wang F, Flanagan J, Su N, Wang LC, Bui S, Nielson A, Wu X, Vo HT, Ma XJ, Luo Y (2012) RNAscope: a novel in situ RNA analysis platform for formalin-fixed, paraffin-embedded tissues. J Mol Diagn 14:22–29. 10.1016/j.jmoldx.2011.08.002 22166544 PMC3338343

[B71] Wolff SB, Gründemann J, Tovote P, Krabbe S, Jacobson GA, Müller C, Herry C, Ehrlich I, Friedrich RW, Letzkus JJ, Lüthi A (2014) Amygdala interneuron subtypes control fear learning through disinhibition. Nature 509:453–458. 10.1038/nature13258 24814341

[B72] Zeisel A, Muñoz-Manchado AB, Codeluppi S, Lönnerberg P, La Manno G, Juréus A, Marques S, Munguba H, He L, Betsholtz C, Rolny C, Castelo-Branco G, Hjerling-Leffler J, Linnarsson S (2015) Cell types in the mouse cortex and hippocampus revealed by single-cell RNA-seq. Science 347:1138–1142. 10.1126/science.aaa1934 25700174

[B73] Zeisel A, Hochgerner H, Lönnerberg P, Johnsson A, Memic F, van der Zwan J, Häring M, Braun E, Borm LE, La Manno G, Codeluppi S, Furlan A, Lee K, Skene N, Harris KD, Hjerling-Leffler J, Arenas E, Ernfors P, Marklund U, Linnarsson S (2018) Molecular architecture of the mouse nervous system. Cell 174:999–1014.e1022. 10.1016/j.cell.2018.06.021 30096314 PMC6086934

[B74] Zeng H, Sanes JR (2017) Neuronal cell type classification: challenges, opportunities and the path forward. Nat Rev Neurosci 18:530–546. 10.1038/nrn.2017.85 28775344

[B75] Zeppilli S, Ackels T, Attey R, Klimpert N, Boeing S, Crombach A, Schaefer A, Fleischmann A (2020) Molecular characterization of projection neuron subtypes in the mouse olfactory bulb. bioRxiv. 2020.2011.2030.405571. 10.7554/eLife.65445 PMC8352594 34292150

